# Mass spectrometry-based multi-omics analysis elucidates immune microenvironmental characteristics and the risk of distant metastasis in N1c colorectal cancer

**DOI:** 10.3389/fimmu.2026.1590042

**Published:** 2026-02-17

**Authors:** Baiwang Zhu, Chenxiao Zheng, Hangjia Xu, Yating Zheng, Yunfei Liu, Peng Li, Haobo Jin, Binbin Ou, Yanyu Chen, Zihan Wang, Qiongying Zhang, Xiaodong Zhang, Yifei Pan

**Affiliations:** 1 Department of Gastrointestinal Surgery, The Third Xiangya Hospital, Central South University, Changsha, China; 2 Department of Colorectal Anal Surgery, The First Affiliated Hospital of Wenzhou Medical University, Wenzhou, Zhejiang, China; 3 Department of Hepatopancreatobiliary Surgery II, The Third Xiangya Hospital, Central South University, Changsha, China; 4 Department of Pathology, The First Affiliated Hospital of Wenzhou Medical University, Wenzhou, Zhejiang, China; 5 Laboratory Animal Centre, Wenzhou Medical University, Wenzhou, Zhejiang, China; 6 General Practice Department, Hangzhou Gongshu Hospital of Integrated Traditional and Western Medicine, Hangzhou, Zhejiang, China; 7 Clinical Medical College, Hangzhou Medical College, Hangzhou, Zhejiang, China

**Keywords:** colorectal cancer, immunotherapy response, metastasis, PLOD1, tumor deposit

## Abstract

**Background:**

Colorectal cancer (CRC) ranks third in global cancer incidence and second in mortality. Tumor deposit (TD), a specific regional spread form, is a crucial independent risk factor for survival and have biological differences from lymph node metastasis (LNM). However, it is underestimated in current staging systems, which results in biased treatment decisions and prognosis evaluation. Moreover, the biological features and distant metastasis patterns of N1c CRC remain largely unknown.

**Method:**

We performed Data-independent Acquisition Mass Spectrometry (DIA-MS) analysis of formalin-fixed, paraffin-embedded (FFPE) samples from 13 T1-T4N1cM1 CRC patients to reveal their molecular characteristics. 9 machine learning algorithms identified 10 TD-related metastasis genes (TDRGs), the multi-cohort validation in 1,582 CRC patients confirmed their role in prognosis. Immune landscape and immunotherapy response were assessed by the CIBERSORT, tumor mutation burden (TMB), Tumor Immune Dysfunction and Exclusion (TIDE) score, Consensus molecular subtype (CMS), immune checkpoint gene expression. scRNA-seq analysis identified Procollagen-Lysine,2-Oxoglutarate 5-Dioxygenase 1 (PLOD1) expression in CRC, Immunohistochemical staining (IHC) and Masson’s trichrome staining were used to assess PLOD1 expression and collagen fiber content in CRC, its role in tumor invasion and migration was elucidated by wound healing and transwell assays.

**Results:**

N1c samples exhibit enhanced extracellular matrix (ECM) remodeling and epithelial mesenchymal transition (EMT). The TDRGs identified by machine learning robustly predicting disease-free survival (DFS) across multiple cohorts (Mean C-index = 0.72) and immune activity. A higher risk score predicted early metastasis and poorer response to immunotherapy, marked by lower level of immune infiltration, higher TIDE scores, lower TMB, and downregulated immune checkpoint genes. scRNA-seq analysis pinpoints highest PLOD1 expression in fibroblasts. Histological analysis of N1c samples demonstrated PLOD1 expression patterns and their significant correlation with stromal collagen fiber abundance (p < 0.01). Wound healing and transwell assays indicating the knockdown of PLOD1 hinders the migration and invasion of CRC DLD-1 cell.

**Conclusion:**

We assessed the protein expression profiles and pathway characteristics of N1c CRC. The model developed based on TDRGs, effectively predicted DFS and immunotherapy response, supporting precision treatment and staging system optimization. PLOD1’s role in ECM remodeling and CRC cell migration and invasion suggests its potential as a prognostic biomarker and therapeutic target.

## Introduction

CRC is currently the third most common cancer globally and the second leading cause of cancer-related deaths, with over 1,800,000 new cases and nearly 900,000 deaths annually worldwide (1). The treatment of CRC heavily relies on the TNM staging system, which is considered the most important prognostic factor for overall survival, metastasis, and recurrence (2).

Beyond the TNM staging system, tumor deposit (TD) has emerged as an independent pathological feature with significant prognostic implications. In AJCC 8th edition, TD are defined as Isolated, discontinuous cancerous nodules located within the lymphatic drainage region of the primary tumor, in the pericolorectal adipose tissue or adjacent mesentery, with no histological evidence of residual lymph nodes or vascular/nervous structures (3). A meta-analysis by Nagtegaal et al. (4) summarized data from five studies and found that TD was observed in approximately 20% of CRC, which were independently associated with worse DFS and OS in multiple cohort studies. They tend to coexist with other pathological features such as lymph node metastasis (LNM), poorer tumor differentiation, perineural invasion, and extramural vascular invasion (EMVI) (5). *Post-hoc* analyses of the IDEA France (6) and CALGB/SWOG 80702 trials (7) found that the negative prognostic impact of TD was observed regardless of pN substage. Recent studies have further found that TD are significantly associated with OS and the risk of distant metastasis, the strong association between TD and poor oncologic outcomes is independent of LNM or neoadjuvant therapy (8).

However, in the current AJCC/TNM8 staging system, TD is only considered in the absence of LNM, and are uniformly classified as N1c stage regardless of their number (3). This classification fails to accurately reflect the complex biological behaviors and prognostic impact of TD, potentially leading to an underestimation of metastasis risk in TD-positive CRC patients and directly influencing adjuvant treatment decisions for stage III colon cancer patients (9).

Unfortunately, to date, the pathogenesis and biological characteristics of TD remain unclear, limiting diagnostic and therapeutic options. Studies suggest that LNM and TD formation are induced by distinct molecular pathways, Fan et al. (10) discovered through immunohistochemical analysis that Snail-induced epithelial-mesenchymal transition (EMT) facilitates LNM, while Twist promotes TD formation via EMT. Another transcriptomic and proteomic study comparing TD and LNM found that TD exhibits a more aggressive, mesenchymal, and fibrotic phenotype. Compared to LNM, TD typically demonstrates increased EMT upregulation, tumor cell migration and invasion, and a more immunosuppressive tumor microenvironment (TME), with higher abundances of fibroblasts and macrophages (11). This highlights the heterogeneity of TD as a form of local regional spread. However, there is still a lack of analysis encompassing the TME and immunology of TD-positive CRC, as well as biological evidence for TD’s role in promoting distant metastasis. Our aim is to further evaluate the immune microenvironment features associated with N1c CRC at the molecular level and identify biomarkers for distant metastasis. This will support further refinement of clinical classifications, definition of relevant patient subgroups, and precision treatment.

In this study, through proteomic analysis of FFPE samples from T1-T4N1cM1 CRC patients, combined with Cytoscape-based gene topology assessment and machine learning algorithms, we identified 10 TDRGs with consistent expression patterns across primary tumors, TDs, and metastases. The prognostic model built on these genes demonstrated robust predictive performance for DFS. Notably, a higher risk score correlated with elevated immune scores yet poorer immunotherapy response, suggesting its role in immune suppression and evasion. Additionally, we observed that TDRG PLOD1 was enriched in fibroblasts and significantly associated with EMT and overexpressed in N1c CRC samples. Consequently, we focused on exploring PLOD1’s role in ECM remodeling and CRC cell migration/invasion, highlighting its potential as a prognostic biomarker and therapeutic target for CRC management.

## Materials and methods

### Patients

**Figure 1:**
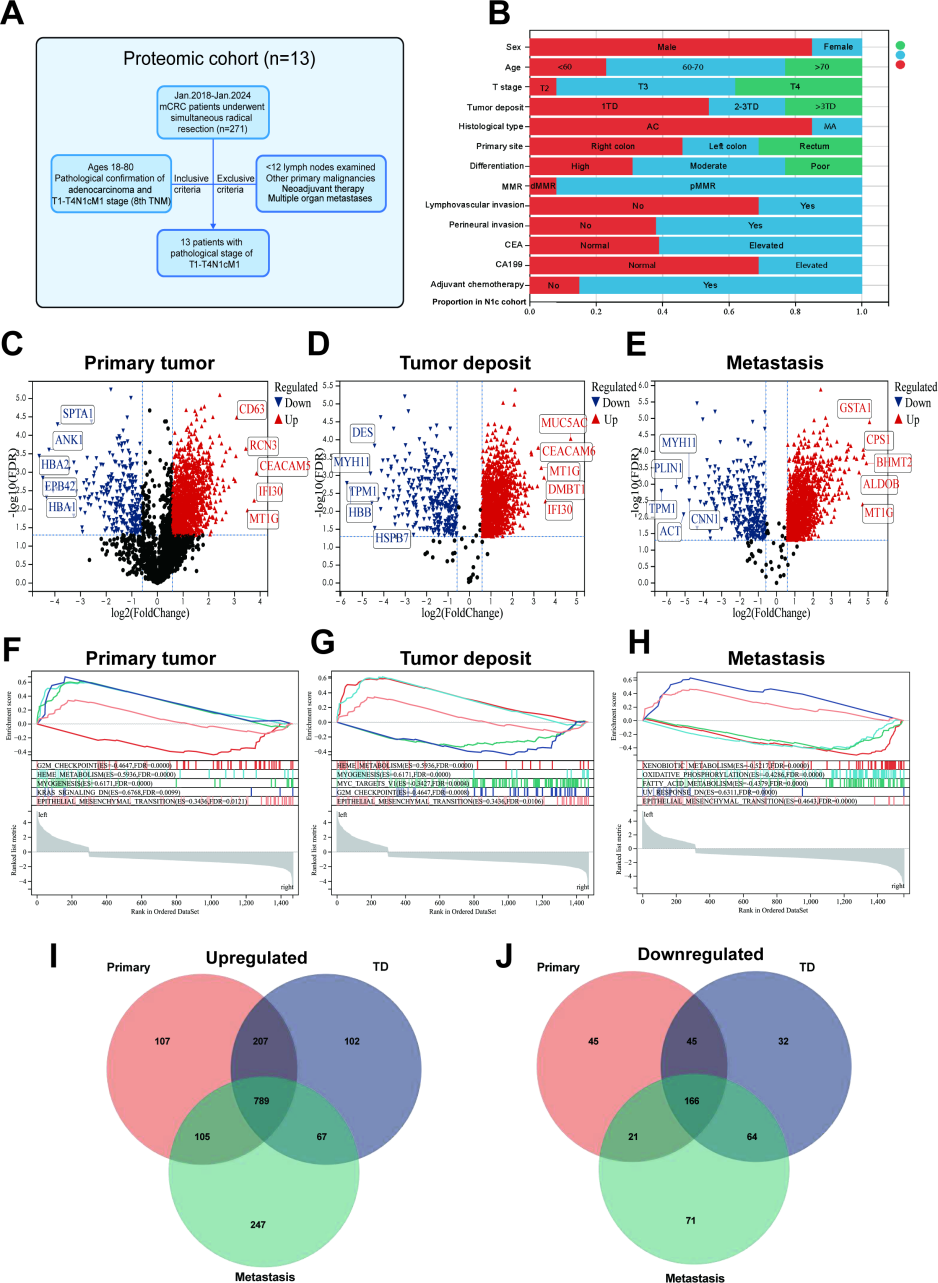
Sample selection and proteomics landscape of T1-T4N1cM1 CRC. **(A)** Flow chart of the selection process. **(B)** Histogram illustrating the distribution of clinicopathological characteristics among patients. Volcano plot of the differential expressed proteins in the primary CRC **(C)**, tumor deposit **(D)**, distant metastasis **(E)** compared to adjacent normal tissues (red/blue dots indicate upregulated/downregulated proteins with FDR<0.05 and fold-change>1.5 or <0.67). Gene set enrichment analysis(GSEA)for the differential expressed protein in primary CRC **(F)**, tumor deposit **(G)**, distant metastasis **(H)**. Venn plot of up-regulated **(I)** and down-regulated **(J)** differential expressed proteins in three types of samples.

The cohort for proteomic analysis was originated from the Colorectal and Anal Surgery Department of the First Affiliated Hospital of Wenzhou Medical University, this study was approved by its Ethics Committee (KY2022-183). We screened 271 mCRC patients who underwent simultaneous radical resection of primary tumors and distant metastases between 2018 and 2024, 13 patients with pathological stage of T1-T4N1cM1 was selected for specimen collection. The selection process is shown in **Figure 1A**. The inclusion criteria were as follows: ages 18-80, receipt of radical surgery, pathological confirmation of colorectal adenocarcinoma and T1-T4N1cM1 stage (8th AJCC/UICC TNM). Exclusion criteria encompassed lymph node metastasis, <12 lymph nodes examined, other primary malignancies, systemic neoadjuvant therapy, and multiple distant metastases to multiple organs. Clinicopathological characteristics are shown in **Figure 1B**. Also, detailed in **Supplementary Table S1**.

### Pathological assessment and sample preparation

**Figure 2:**
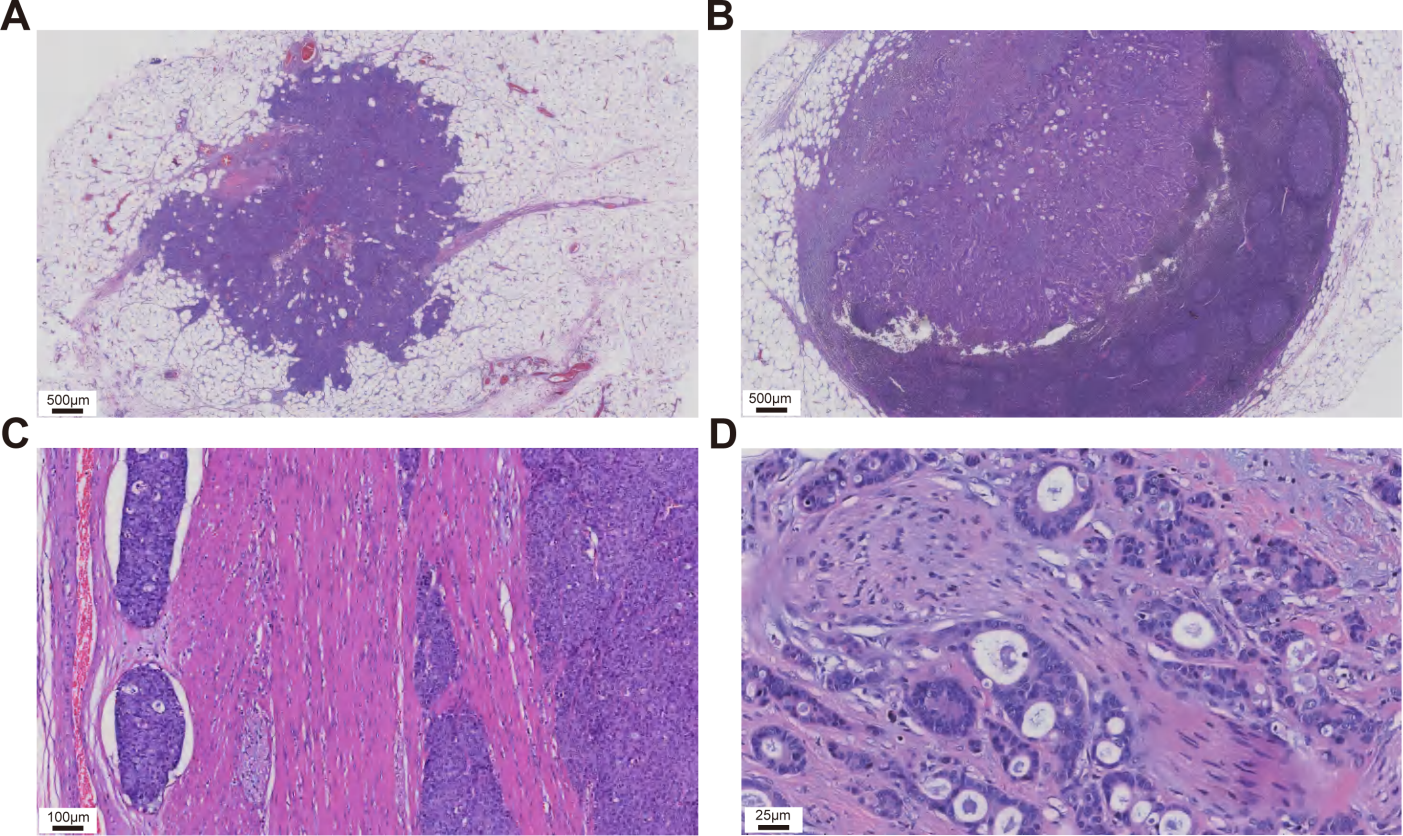
Representative microscopic images illustrating the distinctions between tumor deposits **(A)**, lymph node metastases **(B)**, intravascular tumor deposits **(C)** and perineural tumor deposits **(D)**.

FFPE specimens of normal tissue, primary tumor, TD, and distant metastases were collected from 13 T1-T4N1cM1 CRC patients. Pathological examination by a pathologist confirmed the tumor areas. A multi-step pathological assessment was employed to determine TD status: two pathologists independently reviewed HE-stained FFPE sections using standardized diagnostic criteria based on the AJCC 8th edition TD definition, without access to clinical-pathological data, relying solely on morphological evaluation. A third pathologist adjudicated discrepancies. Representative microscopic images distinguishing TD from lymph node metastasis, as well as intravascular and perineural non-nodular tumor deposits, are shown in **Figure 2**. Inter-observer variability data are provided in **Supplementary Table S2**, with a κ-coefficient of 0.628 indicating substantial agreement. To minimize sample loss, composite samples were prepared by pooling tissues of the same type. Stratified randomization and batch balancing were implemented: samples were stratified by 4 tissue types, and equal protein amounts (10 μg per sample) from 13 patients were randomly pooled into 4 composite samples, which were distributed across different LC-MS batches. Batch randomization was performed, and each composite sample underwent 2 DIA-MS runs to reduce bias.

### Protein extraction and peptide enzymatic digestion

Appropriate amounts of SDT (4% SDS, 100mM Tris-HCl, pH7.6) lysate were added to each sample for protein extraction, and protein quantification was performed using the BCA method. Fifteen micrograms of protein from each sample were mixed with an appropriate volume of 5X loading buffer, boiled for 5 minutes, and then subjected to SDS-PAGE electrophoresis (4%-20% prefabricated gradient gel, constant voltage of 180V for 45 minutes), followed by staining with Coomassie Brilliant Blue R-250. An appropriate amount of protein from all samples was pooled to create a Pool sample, which was used as a QC sample. All samples, including the pooled Pool sample, were digested with trypsin using the Filter-Aided Proteome Preparation (FASP) method. The peptide fragments from the digested samples were desalted using a C18 Cartridge, lyophilized, and then resuspended in 40 μL of 0.1% formic acid solution. The peptide concentration of the samples was determined by OD280 measurement. An appropriate amount of iRT standard peptides were added to the trypsin-digested peptide fragments of each sample, and the samples were subjected to DIA mass spectrometry using an Astral high-resolution mass spectrometer.

### Mass spectrometry assay for DIA

Chromatographic separation for Data-Independent Acquisition Mass Spectrometry (DIA-MS) analysis was performed using the Vanquish Neo system (Thermo Fisher) with nanoliter flow rates. The samples separated by nano-HPLC were subjected to DIA-MS analysis using the Astral high-resolution mass spectrometer (Thermo Scientific). The detection mode was positive ion, with a parent ion scan range of 380–980 m/z. The first-order mass spectrometry resolution was 240,000 at 200 m/z, with a Normalized AGC Target of 500% and a Maximum IT of 5 ms. For MS2, the DIA data acquisition mode was employed with 299 scan windows set up. The Isolation Window was 2 m/z, the HCD Collision Energy was 25 eV, the Normalized AGC Target was 500%, and the Maximum IT was 3 ms. Each composite sample was subjected to two DIA-MS runs, and the inter-replicate correlation was assessed, with an R² value greater than 0.8 considered acceptable.

### Mass spectrometry data analysis

The DIA data were processed using DIA-NN software (1.8.1) with the following parameter settings: trypsin was selected as the enzyme, the maximum number of missed cleavage sites was set to 1, carbamidomethyl (C) was set as the fixed modification, and oxidation (M) and acetyl (Protein N-term) were set as dynamic modifications. Peptide identification was performed directly using the DIA-NN library-free approach. All reported data adhered to a 99% confidence threshold for protein identification, as validated by a false discovery rate (FDR) ≤ 1%. Identified proteins were mapped to corresponding genes using the Universal Protein Resource (UniProt) database to retrieve gene names, functional annotations, and related metadata.

### Quality control

To monitor and evaluate system stability and data reliability, we conducted quality control (QC) sample assessment and DIA workflow validation. A pooled QC sample, containing equal aliquots from all study samples, was inserted at regular intervals throughout the sample cohort, and its consistency was evaluated. QC performance was primarily assessed using the coefficient of variation (CV). For DIA workflow validation, metrics included retention time stability of internal standard calibration peptides (iRT; using an iRT Kit in this study), full width at half maximum (FWHM), and peptide length distribution. Quality control metrics are detailed in **Supplementary Figure S1**.

### Data resources

Target RNA-seq datasets and clinical data (GSE33113, n = 96; GSE38832, n = 122; GSE39582, n = 585) were downloaded as Series Matrix Files from the GEO database (https://www.ncbi.nlm.nih.gov/geo/). After log_2_ transformation, batch effects were corrected using the normalizeBetweenArrays function from the limma R package. The CCRC cohort (n = 146) from Li et al. (12) had undergone preprocessing (log_2_ transformation, median normalization, and batch correction) by the original submitters. TCGA-COADREAD cohort data (n = 626) for Tumor Mutation Burden (TMB) analysis were obtained from The Cancer Genome Atlas (TCGA) portal (https://portal.gdc.cancer.gov/) and normalized using the DESeq2 R package. Differential expression proteins (DEPs) were identified using the limma package (Fold change < 0.67 or >1.5, p < 0.05). Overlaps of DEPs in primary tumors, TDs, and distant metastases were visualized using the “Venn” tool. The CCRC dataset which containing distant metastasis information, was used as the training set, validated by GSE33113, GSE38832, and GSE39582. TCGA-COADREAD were used for mutation analysis, while CRC_EMTAB8107 (n=7) was used for scRNA-seq data analysis.

### Protein-protein interaction network

We utilized the STRING database (http://string-db.org/) to investigate the interaction relationships between target proteins. CytoScape software (version 3.10.0) and GeneMANIA (http://genemania.org/) database generated a protein-protein interaction (PPI) network to identify the co-expression and interactions of key proteins. Utilizing the Molecular Complex Detection (MCODE, version 2.0.3) plugin (https://apps.cytoscape.org/apps/mcode), we extracted potentially tightly connected core gene modules (clusters, subnetworks) within the PPI network.

### Biological function and pathway enrichment analysis

To investigate the biological functions and pathways associated with the DEGs in three types of tissues and the core cluster in the PPI network, we conducted Kyoto Encyclopedia of Genes and Genomes (KEGG), Gene Ontology (GO), and Gene Set Enrichment Analysis (GSEA) using the “ClusterProfiler” package and the GSEA software (https://www.gsea-msigdb.org/gsea/index.jsp). The h.all.v7.4.symbols.gmt subset was downloaded from the Molecular Signatures Database (MSigDB, https://www.gsea-msigdb.org) to assess the correlation between gene expression levels and biological pathways or molecular mechanisms.

### Machine learning identifies TD-related prognostic biomarkers

We integrated nine different machine learning algorithms (13) including Elastic net (Enet), Support Vector Machine (SVM), Least Absolute Shrinkage and Selection Operator (Lasso), Gradient Boosting Machine (GBM), Random Forest (RSF), StepCox, CoxBoost, Super Partial Correlation (Super PC), and Partial Least Squares with Cox Regression (plsRcox). A total of 101 algorithmic combinations were trained on the CRCC cohort to compute the C-index. Feature selection was performed exclusively within the training set to identify 10 core prognostic genes. Hyperparameter optimization and performance evaluation of the model were conducted using 5-fold nested cross-validation (NCV): the inner 5-fold CV employed GridSearchCV for exhaustive parameter tuning, while the outer 5-fold CV assessed model generalization capability. The TDRG model was validated on external datasets (GSE33113, GSE38832, GSE39582) containing DFS information. For each external cohort, individual RiskScore were calculated, and patients stratified into high- and low-risk subgroups based on median score. Prognostic significance was evaluated using Kaplan-Meier (KM) survival analysis. The tidymodels package computed bootstrap-derived Area Under the Curve (AUC), the rmda package performed Decision Curve Analysis (DCA), and the rms package generated Calibration Curves and constructed a nomogram incorporating the TDRG score.

### Immune microenvironment and immunotherapy response

Using the CIBERSORT algorithm (14), we evaluated the abundance of 24 immune cell subsets in high-risk and low-risk samples stratified by TDRGs model. The Tumor Immune Dysfunction and Exclusion (TIDE) score (http://tide.dfci.harvard.edu/) and expression differences of immune checkpoint genes between subgroups predicted immunotherapy response. The “CMSclassifier” package predicts the Consensus Molecular Subtype (CMS) for each sample and displays the proportions among subgroups. The IMvigor210CoreBiologies cohort, comprising sequencing data and clinical survival outcomes from over 200 metastatic urothelial carcinoma patients treated with anti-PD-L1 therapy, was extracted using the IBOR package. We evaluated differences in immunotherapy response between high- and low-risk subgroups (complete response [CR]/partial response [PR] vs. stable disease [SD]/progressive disease [PD]) and analyzed Kaplan-Meier (KM) survival curves using the TDRG model. Correlations between target genes and immune cells were calculated using TIMER (15), QUANTISEQ (16), MCPcounter (17), EPIC (18), and CIBERSORT (14).

### Mutation analysis

Extract mutation data from patients in the TCGA-COAD and TCGA-READ cohorts. Based on the TDRGs model, risk subgroups are classified. Utilize the “mafTools” R package to analyze somatic mutations in the high-risk and low-risk groups, as well as mutations in 10 TDRGs across all samples. Additionally, we analyze the differences in Tumor Mutation Burden (TMB) between the subgroups.

### Drug sensitivity analysis

Based on Cancer Therapeutics Response Portal (CTRP, https://portals.broadinstitute.org/ctrp.v2.1/) and Genomics of Drug Sensitivity in Cancer (GDSC, https://www.cancerrxgene.org/), the “Oncopredict” R package was used to conduct a half-maximal inhibitory concentration (IC50) analysis of drugs for high-risk and low-risk groups of CRC patients.

### Single cell RNA sequencing analysis

We acquired a CRC dataset (CRC_EMTAB8107) from the Tumor Immune Single Cell Hub 2.0 (TISCH 2.0) database (https://tisch.compbio.cn/) (19), comprising 23,176 cells from 7 tumor samples. Subsequent analyses included scRNA-seq for the PLOD1 and Cell-Cell Interaction (CCI) analysis and visualize the expression and distribution of PLOD1, and the interactions between target gene-enriched cell subpopulations and others.

### Antibodies, plasmids, cell lines and culture

The following two antibodies were used in this study: PLOD1 (#29480-1-AP, Proteintech, USA) and GAPDH (#2118, Cell Signaling Technology, USA). The knockdown plasmid for PLOD1 was synthesized by Miaoling Bioscience (Wuhan, China). The DLD-1 (#CBP60037) cell line was obtained from the Chinese Academy of Sciences (CAS) and authenticated by STR profiling. The cells were cultured in RPMI 1640(#C11875500BT, Gibco, USA) and maintained in a humidified incubator with an atmosphere of 5% CO_2_. The medium was supplemented with 10% FBS and 10,000 U/ml penicillin-streptomycin (#15140122, Gibco, USA).

### Immunohistochemical assay

The tissue specimens were fixed in 4% paraformaldehyde, subsequently embedded in paraffin, and sectioned into 4 µm thick slices for slide preparation. Following gradient deparaffinization and rehydration, antigen retrieval was performed using a microwave method with citrate buffer (100 °C, four cycles, 7 minutes per cycle). The slides were then thoroughly washed with PBS. A blocking step was conducted for 30 minutes to prevent nonspecific binding. The primary antibody was incubated at 4 °C overnight, followed by incubation with the secondary antibody at room temperature. Color development was achieved using DAB chromogen, and the sections were counterstained with hematoxylin. To quantitatively analyze PLOD1 gene expression, this study utilized Image-Pro Plus 6.0 to capture at least five non-overlapping fields of view (FOVs) per tissue section under 400× magnification. The integrated optical density (IOD) and area of PLOD1 protein expression were measured in each FOV, with statistical comparisons performed using the mean IOD/Area values across experimental groups.

### Masson trichrome staining assay

FFPE tissue section slides were deparaffinized and rehydrated, then stained using the Masson Trichrome Staining Kit (#G1006, Servicebio, China). Sections were mordanted in Masson A solution at 65°C for 30 minutes, rinsed with running water, and stained with a fresh 1:1 mixture of Masson B and C solutions for 1 minute, followed by 1% hydrochloric acid-ethanol differentiation until nuclei appeared gray black against a nearly colorless background. After brief rinsing, sections were immersed in prewarmed Masson D solution for 6 minutes (3 minutes for frozen sections) to stain muscle fibers red, then rinsed until colorless. Brief differentiation in Masson E solution optimized contrast before transfer to prewarmed Masson F solution for collagen staining (duration adjusted from seconds to 1 minute by tissue type). Sections were then rapidly rinsed in 1% glacial acetic acid to remove excess blue dye under microscopic control, dehydrated in absolute ethanol, cleared in xylene, and mounted with neutral balsam. Staining results showed blue collagen fibers, red muscle fibers/cytoplasm, and dark blue-black nuclei. Fibrosis quantification was performed using ImageJ by capturing ≥5 non-overlapping 400× fields per section, applying color thresholding to calculate blue-stained collagen area (Area_Collagen) and total tissue area (Area_Total), with fibrotic content (%) = (Area_Collagen/Area_Total) × 100. Statistical comparisons used mean collagen volume fraction (CVF) values across groups.

### Wound healing assay

Cells were plated in 6-well plates and grown to confluence. A sterile pipette tip scratched the monolayer, which was then washed with PBS to remove any dislodged cells. Culture medium with 1% (fetal bovine serum) FBS was added. Images of cell migration were taken at 0-, 24-, and 48-hours post-wounding. The wound closure area was calculated as: Migration Area (%) = (X0 - Xn)/X0 × 100, where X0 is the initial wound area and Xn is the area at a specific time.

### Transwell assay

The invasive and metastatic potential of DLD-1 cells was assessed using a Matrigel-coated Transwell assay. Briefly, 3×10^4 cells were seeded in the upper chamber of a Transwell with serum-free medium, while the lower chamber contained 10% FBS-supplemented medium. After 24 hours of incubation at 37 °C, cells in the upper chamber were fixed with methanol and stained with Giemsa for quantitative microscopic analysis of invasion and migration.

### Western blot assay

Cell proteins were extracted with a lysis buffer (10 mM TRIS-HCL, pH 7.4, 1% SDS, 1 mM Na3VO4), then lysed ultrasonically and quantified using a microspectrophotometer. After mixing with loading buffer and a marker, samples were loaded onto an 8% SDS-PAGE gel and electrophoresed at 80V for 30 min and 120V for 90 min. Proteins were then transferred to a PVDF membrane (25V, 120 min) and incubated with blocking buffer at 4°C for 3 hours, followed by overnight incubation with primary antibodies at 4°C and 3 hours with secondary antibodies. Bands were visualized using an ECF developer (RPN5785, GE Healthcare) and captured with a chemiluminescent imaging system (GE Healthcare).

### Statistical analysis

All statistical analyses were conducted using R software (version 4.4.2). The Wilcoxon test compared variables between groups, the Chi-square test assessed categorical variable differences, Pearson correlation analyzed variable correlations, and Kaplan-Meier survival analysis with log-rank test evaluated differences. For multiple hypothesis testing, the Benjamini-Hochberg (BH) procedure was applied to control the false discovery rate (FDR), with significance thresholds annotated as follows: *FDR < 0.05; **FDR < 0.01; ***FDR < 0.001; NS (not significant). Unadjusted statistical significance was defined as: *p < 0.05; **p < 0.01; ***p < 0.0001; NS (not significant).

### Ethics approval

This study was conducted in accordance with the ethical standards outlined in the Helsinki Declaration II and certified by the Ethics Committee of The First Affiliated Hospital of Wenzhou Medical University (KY2022-183). Given the retrospective nature of the study, informed consent was waived.

## Results

### Proteomic characteristics of T1-T4N1cM1 patients

To identify the protein landscape and pathways changes in T1-T4N1cM1 CRC, we used normal tissue as a control and analyzed the differentially expressed proteins (DEPs) in primary tumor, TD, distant metastasis samples and conducted a comprehensive comparison of biological pathways and functions. Differential analysis revealed 1,208 upregulated and 277 downregulated proteins in primary tumors (**Figure 1C**), 1,165 upregulated and 307 downregulated in TD (**Figure 1D**), and 1,238 upregulated and 321 downregulated in distant metastasis samples (**Figure 1E**). GSEA was performed on proteins with significant expression differences across three types of samples using the h.all.v7.4.symbols.gmt subset from MSigDB. Results showed enrichment in G2M Checkpoints, Heme metabolism, Myogenesis, KRAS signaling, and EMT in primary tumors (**Figure 1F**). DEPs in TD showed enrichment in Heme metabolism, Myogenesis, MYC targets V1, G2M Checkpoints, and EMT (**Figure 1G**). Enrichment in Xenobiotic metabolism, Oxidative phosphorylation, Fatty acid metabolism, UV response DN, and EMT were showed in distant metastases (**Figure 1H**). Analysis of GO and KEGG pathway roles of these proteins is provided in **Supplementary Figure S2**. Then, Venn diagrams visualized the intersections of up-regulated (**Figure 1I**) and down-regulated (**Figure 1J**) DEPs among primary tumor, TD, and distant metastases. Through GSEA, we observed sustained activation of the G2M checkpoint, EMT pathway, and KRAS signaling across diverse tumor tissue types, providing insights into the molecular mechanisms underlying tumor progression in N1c CRC patients.

### Construction of metastasis-driving protein modules and core pathway analysis

**Figure 3:**
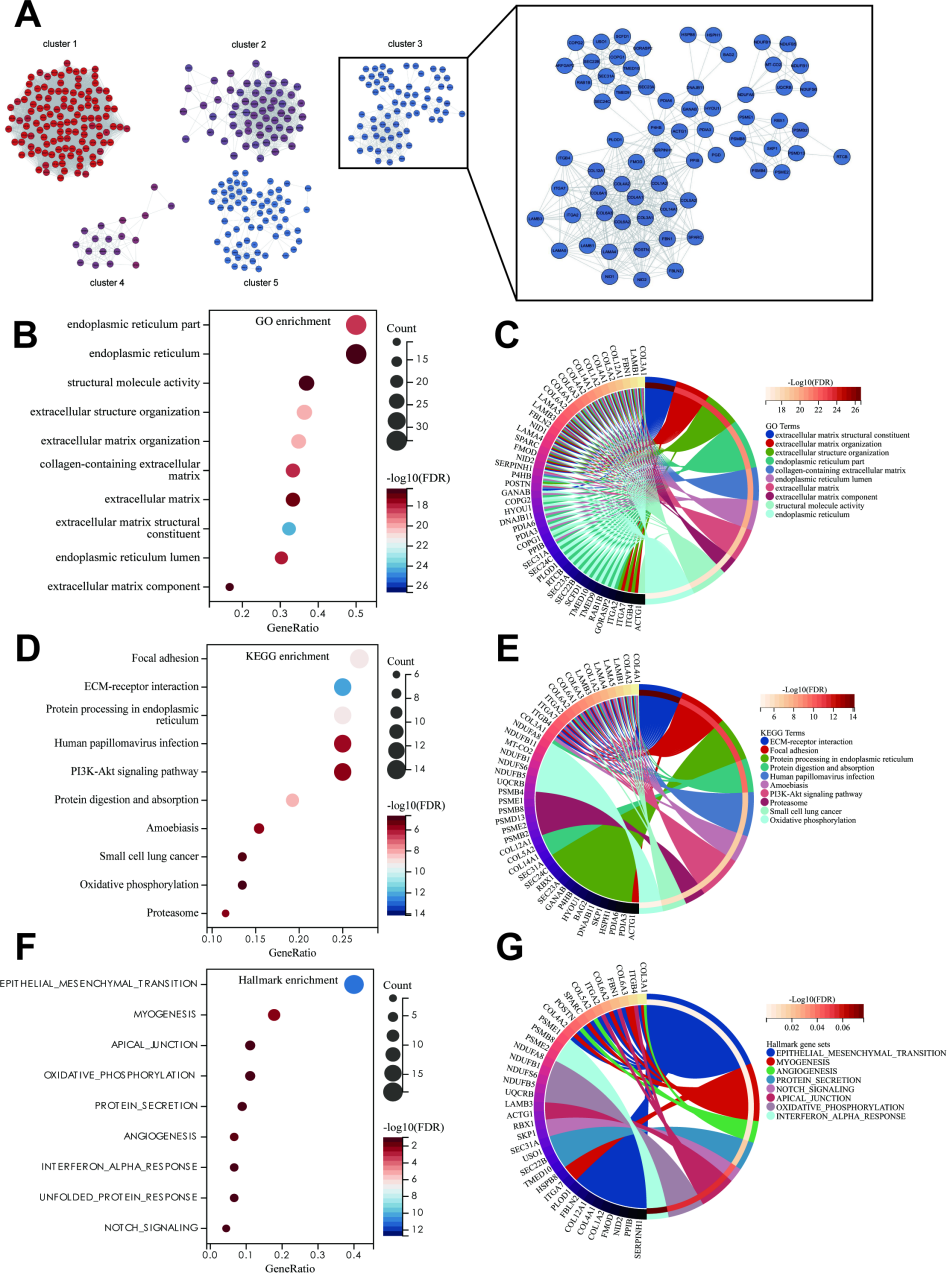
Preliminary screening of core biomarkers associated with TD-related metastasis. **(A)** MCODE in Cytoscape identified a core module consisting of 67 nodes from the PPI network. The GO **(B)**, KEGG **(D)** and hallmark **(F)** enrichment analysis of the 67 genes from the identified cluster3. Chord diagrams of GO **(C)**, KEGG **(E)**, and hallmark **(G)** enrichments show associations of 67 genes across different biological aspects.

We input the 1,086 DEPs that are common to both the primary tumor and distant metastasis into the STRING database to construct a PPI network. Visualization with Cytoscape software and MCODE plugin identified the top five modules (**Supplementary Table S3**). We uncovered a dense regulatory network of ECM remodeling and EMT-related proteins in Cluster3, with 67 nodes and 433 edges (**Figure 3A**). GO analysis reveals that this subnetwork is mainly enriched in extracellular matrix, endoplasmic reticulum, collagen-containing extracellular matrix, and structural molecule activity (**Figures 3B, C**). KEGG analysis primarily focuses on ECM-receptor interaction, Focal adhesion, Protein processing in endoplasmic reticulum, Protein digestion and absorption, Human papillomavirus infection, Amoebiasis, PI3K-Akt signaling pathway, Proteasome, Small cell lung cancer, and Oxidative phosphorylation (**Figures 3D, E**). Hallmark pathway enrichment analysis shows enrichment in EMT pathway, myogenesis, angiogenesis, protein secretion, notch signaling, apical junction, oxidative phosphorylation, and interferon alpha response (**Figures 3F, G**).

### Machine learning-driven prognostic model construction and clinical validation

**Figure 4:**
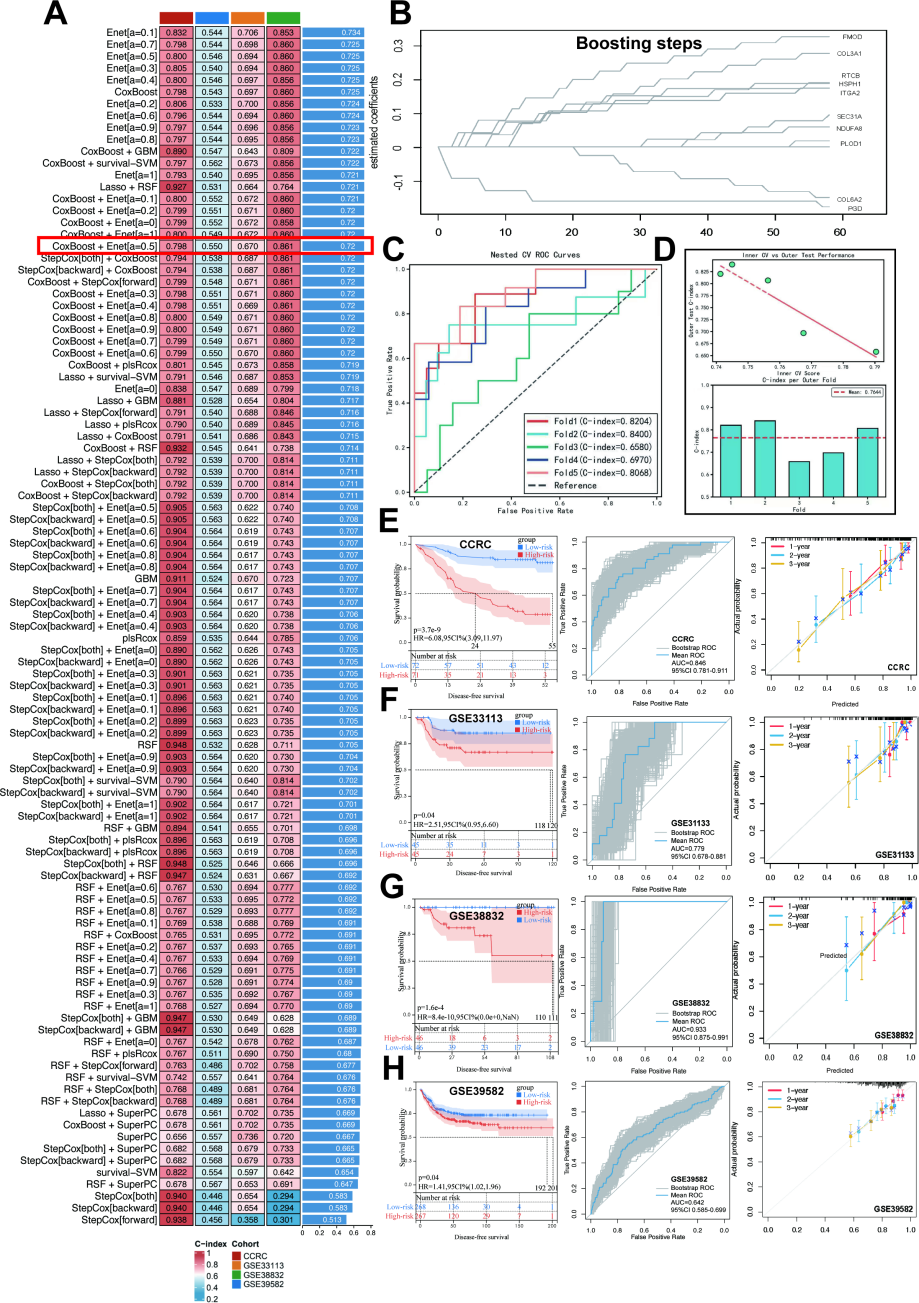
Machine learning identifies TDRGs signature and establishes the best prognostic model. **(A)** 101 different combinations of 9 machine learning algorithms, each model’s c-index across cohorts was calculated. **(B)** Coxboost identified 10 TDRGs with the highest prognostic value. In the CCRC training set, the Outer Test C-index ROC **(C)** and NCV Performance Plot **(D)** were obtained through 5-Fold NCV of the Enet model. For the developed TDRG model, K-M curves, Bootstrap ROC, and calibration curves for DFS prediction were generated in CCRC cohort **(E)**, GSE33113 **(F)**, GSE38832 **(G)**, GSE39582 **(H)**.

**Figure 5:**
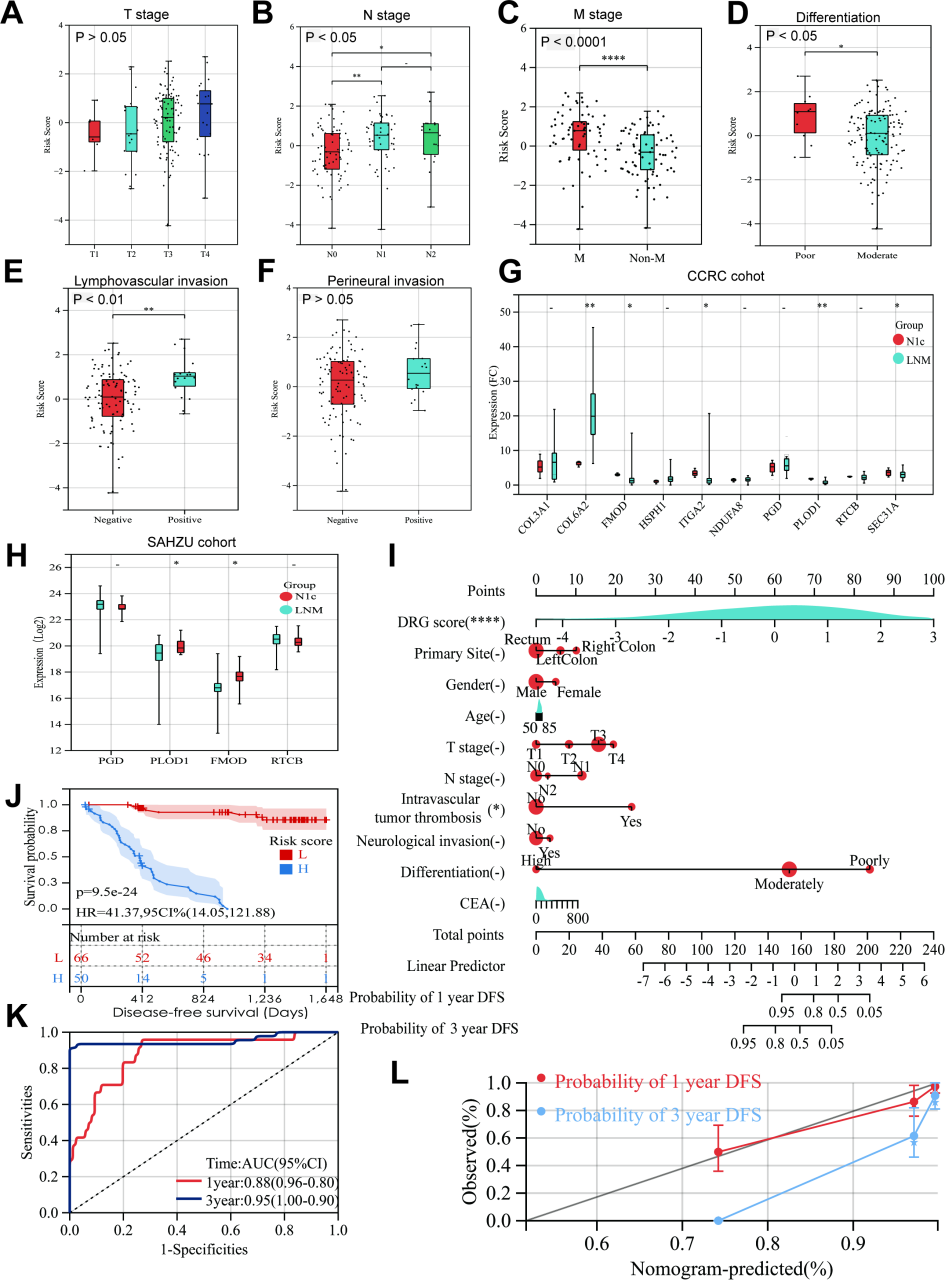
Correlation between the TDRGs and clinicopathological features. **(A)** Correlation between TDRGs and T stage. **(B)** Correlation between TDRG and M stage. **(C)** Correlation between TDRGs and tumor differentiation. **(D)** Correlation between TDRGs and lymphovascular invasion. **(E)** Correlation between TDRGs and perineural invasion. The differential expression of TDRGs between N1c and LNM CRC patients across CCRC cohort **(F)** and SAHZU cohort **(G)**. **(I)** A nomogram for predicting DFS was constructed using multivariate Cox regression. **(J–M)** curves were generated to compare high- and low-risk groups stratified by median risk score. The accuracy of this nomogram in predicting 1-year and 3-year DFS was evaluated by ROC curves **(K)** and calibration curves **(L)**. * p < 0.05, ** p < 0.01, **** p < 0.0001.

Using UniProt protein-to-gene mapping, we obtained gene names and related information for DEPs in Cluster 3. A total of 101 machine learning algorithms—including Enet, SVM, Lasso, GBM, RSF, StepCox, CoxBoost, Super PC, plsRcox, and their combinations—were applied to the training set for feature selection to identify prognostic genes and construct predictive models (**Figure 4A**). 5-fold NCV was employed to assess model stability. In the training cohort CCRC, the CoxBoost+Enet algorithm selected 10 genes (**Figure 4B**: PLOD1, FMOD, ITGA2, COL3A1, COL6A2, HSPH1, SEC31A, PGD, RTCB, NDUFA8), which were used to build the TDRG model. Five-fold NCV validated the Enet model, with ROC curves of C-indices across folds (**Figure 4C**) and NCV performance plots (**Figure 4D**) demonstrating model reliability. External validation was performed on independent datasets, where patients in each cohort were stratified into high- and low-risk groups based on median risk scores. K-M analysis of DFS, bootstrap ROC curves and calibration plots of CCRC (**Figure 4E**), GSE33113 (**Figure 4F**), GSE38832 (**Figure 4G**) and GSE39582 (**Figure 4H**) along with the DCA curves of each cohort (**Supplementary Figure S3**) further emphasized the model’s predictive accuracy. Additionally, we explored correlations between TDRGs and pathological features. Further evaluation reveals significant associations between the RiskScore and advanced N stages (**Figure 5B**) and M stage (**Figure 5C**), poor tumor differentiation (**Figure 5D**), and the presence of intravascular tumor thrombus (**Figure 5E**) (all p<0.05). Additionally, no significant differences in Riskscore were observed across T stages (**Figure 5A**), neurological invasion statuses (**Figure 5F**), and tumor locations (**Supplementary Figure S4A**), MMR, KRAS, BRAF, TP53 mutation statuses (**Supplementary Figures S3B–E**). Given the specificity of the N1c stage, we compared TDRGs expression between N1c and LNM patients in CCRC cohort (**Figure 5G**) and the study cohort SAHZU of Xu et al. (20) (**Figure 5H**). The results showed differential expression of COL6A2, ITGA2, PLOD1, FMOD and SEC31A.To integrate the TDRG score with key clinicopathological covariates, we developed a nomogram based on a multivariate Cox proportional hazards model for predicting 3-year DFS (**Figure 5I**). The TDRG score retained independent prognostic significance in this model (p < 0.0001). The K-M curves for subgroups divided by the median score in the nomogram demonstrated its prognostic value (**Figure 5J**). Additionally, we provided the results of ROC analysis (**Figure 5K**) and calibration curves (**Figure 5L**) to demonstrate its reliability. This nomogram, which combines the TDRG score with the covariates, offers a molecular tool for risk stratification in CRC patients.

### TDRGs correlate with the immune microenvironment and immunotherapy response

**Figure 6:**
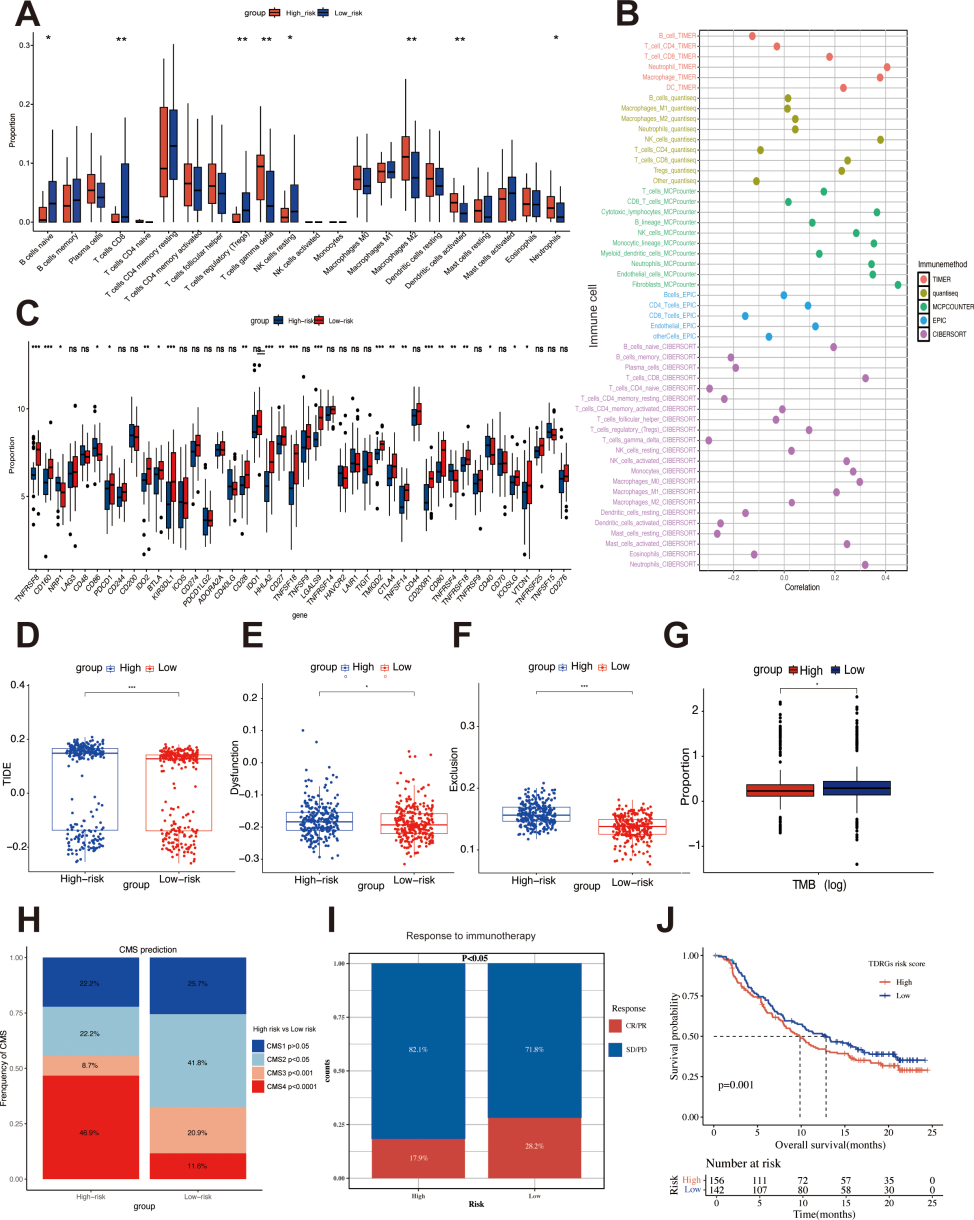
Assessment of immune infiltration and response to immunotherapy in different risk groups. **(A)** Differences in immune cell infiltration among different risk groups. **(B)** Analysis of the correlation between PLOD1 expression and immune cells. **(C)** Differences in the expression levels of immune checkpoint genes among different risk groups. TIDE analysis **(D)** including comparison of Dysfunction score **(E)** and Exclusion score **(F)** across risk groups. **(G)** TMB analysis between high-risk and low-risk groups. **(H)** CMS prediction across risk groups. Differences in immunotherapeutic responsiveness **(I)** and K-M analysis results for OS **(J)** between patients with high and low TDRG scores in the IMvigor210CoreBiologies cohort. * p < 0.05, ** p < 0.01, *** p < 0.001.

Given the significance of the tumor immune microenvironment, TIDE score, CMS subtype, immune checkpoint gene expression, and TMB in immunotherapy response and prognosis, we evaluated these factors based on risk groups determined by the TDRGs score. Immune infiltration analysis by CIBERSORT revealed that the high-risk group had lower proportions of naive B cells, CD8 T cells, Tregs, and resting NK cells, but higher proportions of gamma delta T cells, M2 macrophages, activated dendritic cells, and neutrophils (**Figure 6A**). Furthermore, the positive correlation between TDRG PLOD1 expression and various immune cells was calculated by five algorithms (TIMER, QUANTISEQ, MCPcounter, EPIC, CIBERSORT) (**Figure 6B**), specifically with high levels of CAFs. This correlation may contribute to the poor prognosis in CRC patients with high PLOD1 expression. Analysis of immune checkpoint expression showed most immune checkpoint genes, such as PDCD1 and CTLA4, were expressed at lower levels in the high-risk group (**Figure 6C**). The high-risk group also exhibited a significantly higher TIDE score (**Figure 6D**), with higher T cell Dysfunction score (**Figure 6E**) and Exclusion scores (**Figure 6F**), additionally, the high-risk group had a lower TMB (**Figure 6G**). CMS prediction revealed a significantly higher proportion of CMS4 in the high-risk group (**Figure 6H**) that may suggest upregulated EMT and a high level of CAFs infiltration in the tumor stroma of high-risk patients.

To mitigate the limitations of relying solely on biological predictors of immunotherapy response, we analyzed the IMvigor210CoreBiologies cohort using the TDRGs model to compare proportions of complete/partial response (CR/PR) versus stable/progressive disease (SD/PD) between high- and low-risk subgroups. The results (**Figure 6I**) demonstrated a significantly higher CR/PR rate in the low-risk group (28.2% vs. 17.9%, p < 0.05). Furthermore, K-M analysis (**Figure 6J**) revealed superior OS in the low-risk cohort (p < 0.01), supporting the hypothesis that heightened immune exclusion in high-risk patients contributes to poorer immunotherapy responses.

While analyzing TME heterogeneity, we further explored mutation patterns across risk groups.

we examined mutation patterns across risk groups. Somatic mutation profiling in the TCGA-COADREAD cohort revealed significant enrichment of APC, TP53, TTN, and KRAS mutations across risk subgroups, with APC showing the highest mutation frequency (**Supplementary Figures S5A, B**). Notably, COL3A1 and COL6A2 mutations specifically accumulated in TDRGs (**Supplementary Figure S5C**), mutation of these ECM-related gene may alter tumor stroma composition and impair immune cell infiltration.

Consistent with these findings, the high-risk group exhibited reduced immune cell infiltration, lower immune checkpoint gene expression, elevated TIDE scores, and reduced TMB. When integrated with immunotherapy response analyses from external cohorts, these features collectively indicate compromised immune function and poorer immunotherapy responsiveness.

### Drug sensitivity analysis and therapeutic translation of TDRGs

**Figure 7:**
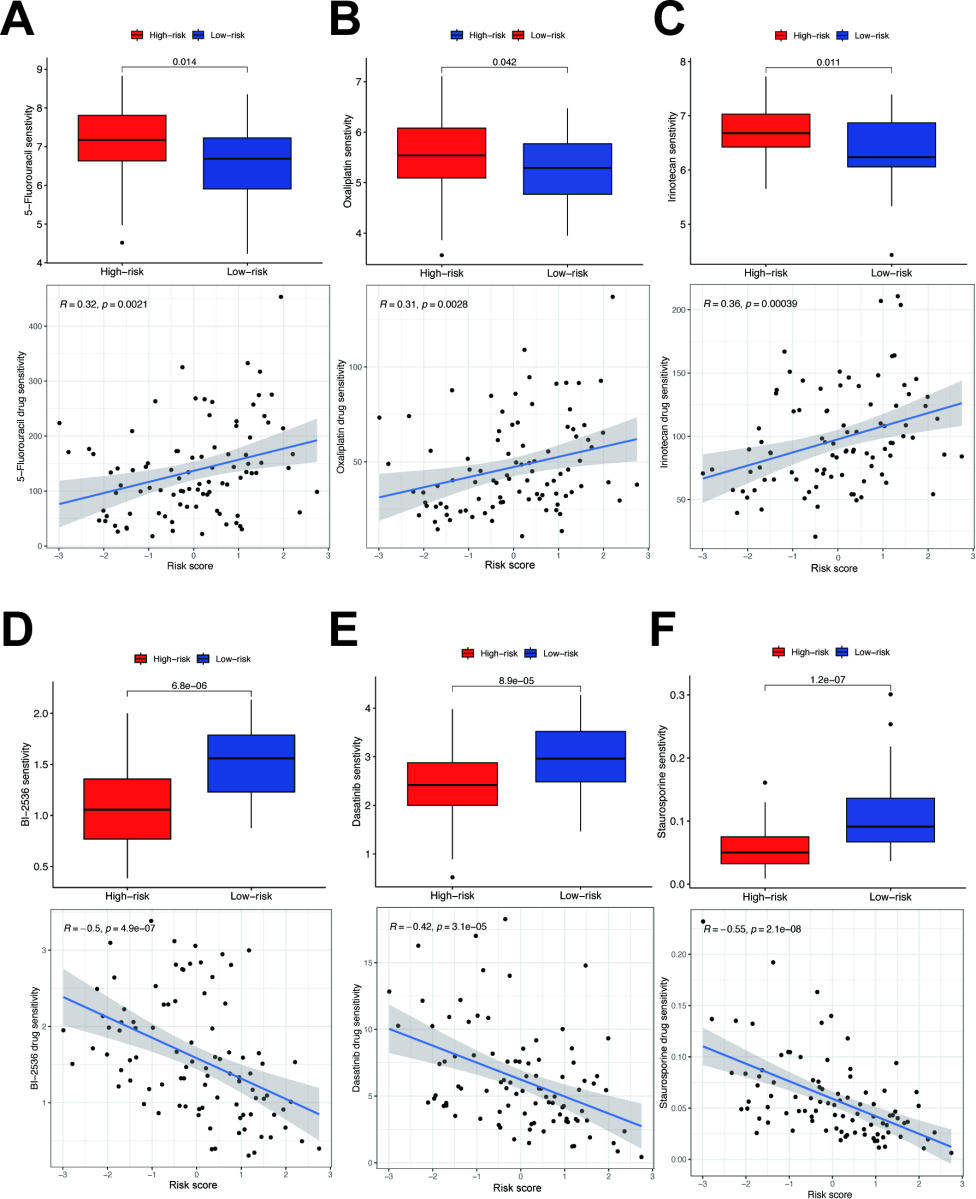
Drug sensitivity analysis in different risk groups. **(A)** Sensitivity analysis of 5-fluorouracil. **(B)** Sensitivity analysis of oxaliplatin. **(C)** Sensitivity analysis of irinotecan. **(D)** Sensitivity analysis of PLK1 inhibitor BI2536. **(E)** Sensitivity analysis of Dasatinib. **(F)** Sensitivity analysis of Staurosporine.

To predict drug sensitivity and identify potential therapeutic drugs for high-risk CRC patients, we calculated IC50 values for three commonly used CRC chemotherapy drugs (5-Fluorouracil, Oxaliplatin, Irinotecan) in different risk subgroups using “Oncopredict” R package and assess the correlation between risk scores and drug sensitivity. The results showed that high-risk patients had poorer sensitivity to 5-Fluorouracil (**Figure 7A**), Oxaliplatin (**Figure 7B**), and Irinotecan (**Figure 7C**), with IC50 values positively correlated with risk scores. Conversely, high risk patients exhibited higher sensitivity to PLK1 inhibitor BI2536 (**Figure 7D**), Dasatinib (**Figure 7E**), Staurosporine (**Figure 7F**), with IC50 values negatively correlated with risk scores. Predictions derived from cell line models suggest that BI2536, dasatinib, and staurosporine may hold potential for overcoming chemotherapy resistance in high-risk CRC patients. Future validation using patient-derived tumor xenograft (PDX) models or organoid systems is warranted to assess clinical translational potential.

### Identification of the interaction network between TDRGs and EMT and the regulatory factor PLOD1

**Figure 8:**
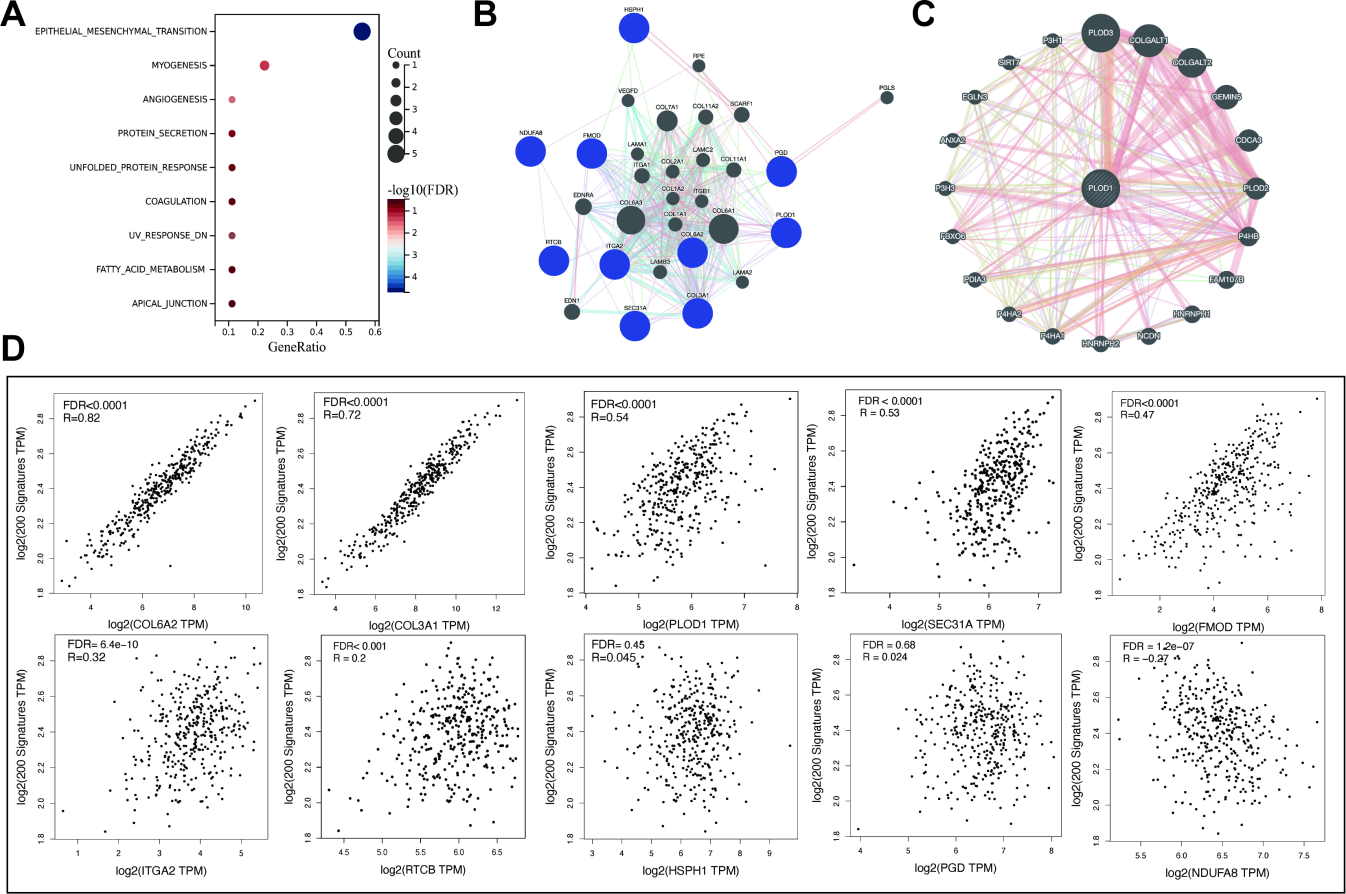
The enrichment and interaction analysis of TDRGs and correlation with the EMT pathway. **(A)** Hallmark enrichment analysis of TDRGs. **(B)** Interactions among TDRGs. **(C)** The PPI network centered on PLOD1. **(D)** Correlation analysis of TDRGs genes with the EMT pathway gene set.

The hallmark enrichment revealed that TDRGs are mainly enriched in the EMT pathway (**Figure 8A**). The results of GO and KEGG enrichment analyses of TDRGs are shown in **Supplementary Figure S6**. EMT is considered a crucial step in tumor metastasis and malignant development. We investigated the correlation between TDRGs and the EMT pathway. 200 EMT-related genes were obtained from the MSigDB database v7.1 and we analyzed their correlation with TDRGs using Pearson correlation analysis. COL6A2, COL3A1, and PLOD1 showed the strongest correlation with the EMT pathway (**Figure 8D**), suggesting these genes may form a core module regulating EMT. Further analysis using the GeneMANIA-built TDRGs interaction network (**Figure 8B**) and the PLOD1-centered PPI network (**Figure 8C**) confirmed that PLOD1, as a central node, links ECM remodeling proteins like collagen post-translational modification enzymes, indicating its potential role in promoting metastasis by ECM remodeling and collaborating with EMT. For more classic EMT-related genes, we conducted differential expression analysis across risk groups in multiple cohorts (GSE38832, GSE31133), the results revealed significant differences in CDH, FOXC2, TWIST1, TWIST2, SNAI2, ZEB1, and ZEB2 (**Supplementary Figure S7**), reinforcing the functional linkage between TDRGs and EMT regulation, providing mechanistic insights into their collaborative roles in tumor progression.

### Single-cell expression profiling of PLOD1

**Figure 9:**
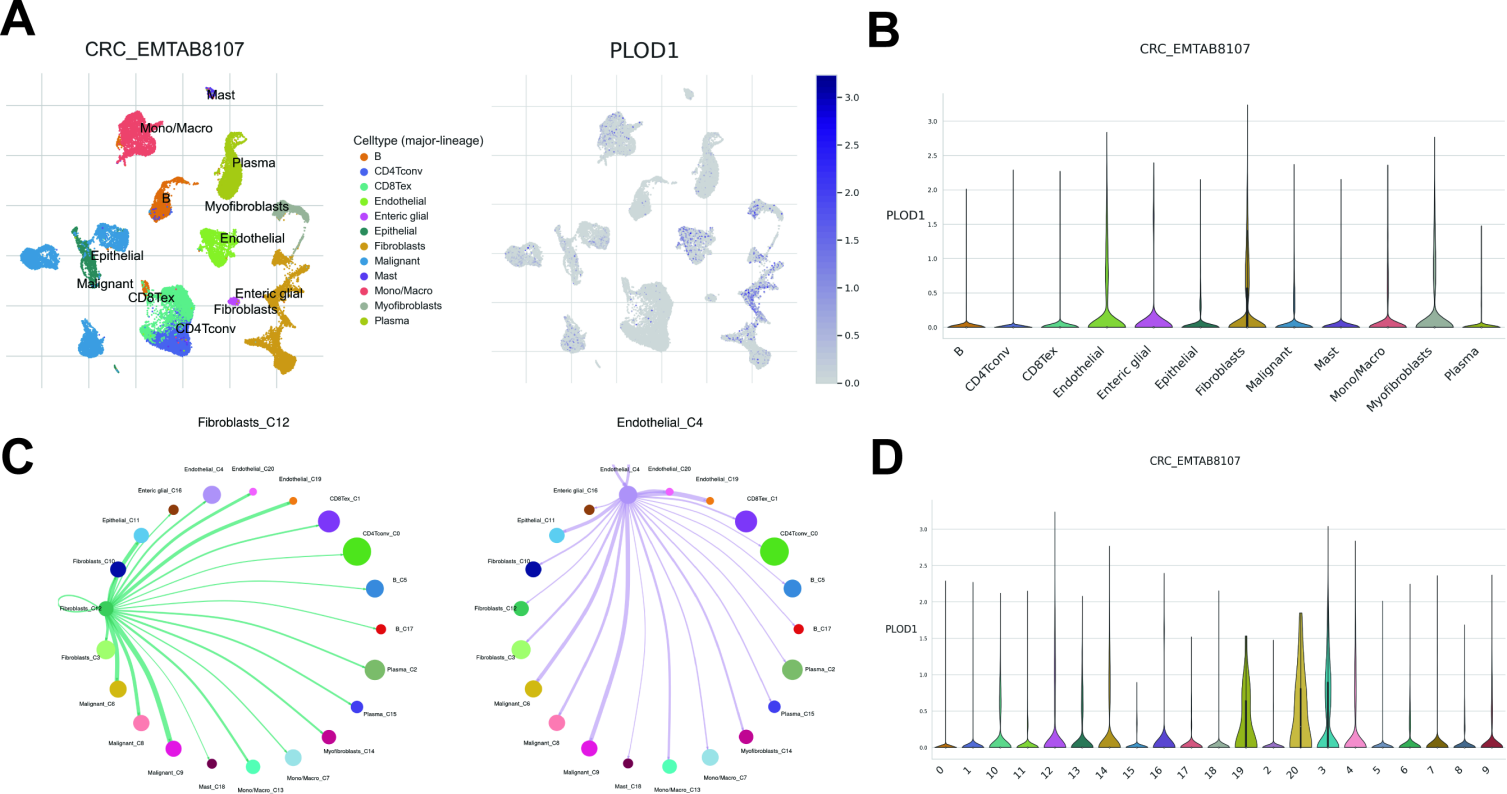
Single cell RNA sequencing analysis. **(A)** UMAP projection of all cells and PLOD1 expression from CRC_EMTAB8107. **(B)** Expression distribution of PLOD1 across different cell types. **(C)** CCI analysis centered on Fibroblasts_C12 and Endothelial_C4. **(D)** Expression distribution of PLOD1 across different cell clusters.

To localize PLOD1 to specific cell subsets and elucidate its TME basis as an EMT regulator, we conducted scRNA-seq analysis using the CRC_EMTAB8107 dataset from TISCH 2.0 database. We identified 20 cell clusters and 12 cell types within CRC tissues (**Figure 9A**). We also observed a significant enrichment of PLOD1 in CAFs and TECs (**Figure 9B**), especially within Fibroblasts_C12 and Endothelial_C4 (**Figure 9D**). The analysis of Cell-Cell Interactions (CCI) revealed that both the Fibroblasts_C12 and Endothelial_C4 mainly interacted with malignant cell (**Figure 9C**).

### Tissue-level validation of PLOD1 expression and its correlation with ECM

**Figure 10:**
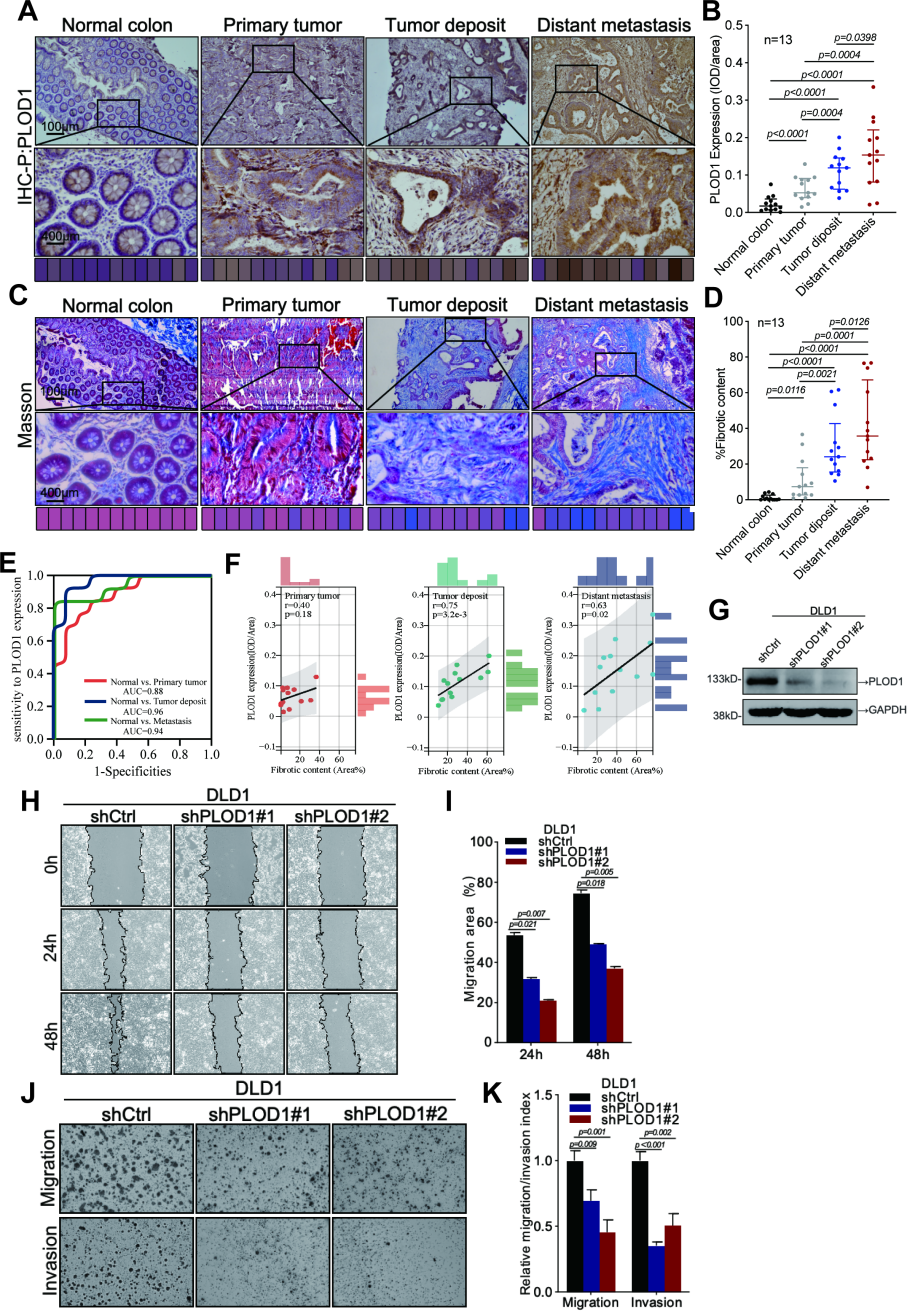
PLOD1-Related Analyses: IHC, Masson’s Trichrome Staining, and Cellular Experiments. **(A)** IHC analysis of PLOD1 expression in normal colon tissues, primary tumors, tumor deposits, and distant metastases. **(B)** Wilcoxon test revealed significant differences in PLOD1 expression among tissues (t-test, p < 0.05). **(C)** Masson’s trichrome staining analysis of different tissue types from patients. **(D)** Wilcoxon test demonstrated significant differences in collagen fiber content among tissues (t-test, p < 0.05). **(E)** ROC curve analysis indicated that PLOD1 expression effectively discriminates between normal and tumor tissues. **(F)** Pearson correlation analysis of PLOD1 protein expression levels with the proportion of collagen fiber. **(G)** Stable knockdown of PLOD1 in DLD1 cells. **(H, I)** Wound healing assays using PLOD1-knockdown cells to assess cell migration capacity. **(J, K)** Transwell assays with PLOD1-knockdown cells to evaluate cell invasion potential.

To verify tissue-specific PLOD1 expression and its association with ECM remodeling in N1c CRC, IHC staining was performed on paired tissue samples from 13 N1c CRC patients, encompassing adjacent normal tissue, primary tumors, TDs, and distant metastases. The results revealed a progressive increase in PLOD1 expression across these tissue types, with statistically significant differences between groups (**Figures 10A, B**; t-test, all *p* < 0.05). ROC curve analysis demonstrated PLOD1’s diagnostic efficacy in distinguishing malignant from normal tissues (**Figure 10E**), suggesting its potential as a CRC biomarker.

Given PLOD1’s role in collagen crosslinking, Masson’s trichrome staining was utilized to quantify collagen fiber abundance in N1c tissues. Collagen content exhibited a stepwise elevation from adjacent normal tissue to primary tumors, TD, and distant metastases, with significant intergroup variations (**Figures 10C, D**; t-test, *p* < 0.05). Pearson correlation analysis further revealed strong positive associations between PLOD1 expression and stromal collagen density in TD (*r* = 0.75, *p* < 0.001) and distant metastases (*r* = 0.63, *p* = 0.02) (**Figure 10F**), supporting PLOD1’s potential role in facilitating CRC metastasis through ECM remodeling. These findings, derived from a limited sample size, require further confirmation through *in vitro* collagen crosslinking experiments and animal models.

### Cellular functional validation of PLOD1

To elucidate PLOD1’s cellular functions, stable PLOD1 knockdown was achieved in DLD-1 CRC cells via shRNA transfection, with knockdown efficiency confirmed by Western blotting (**Figure 10G**). Wound healing and Transwell assays were conducted to assess the impact of PLOD1 depletion on cell migration and invasion. The results indicated that PLOD1 knockdown significantly suppressed DLD-1 cell migration (**Figures 10H, I**; t-test, all *p* < 0.05) and invasion (**Figures 10J, K**; t-test, all *p* < 0.05).

## Discussion

CRC is a complex and heterogeneous disease, the management and treatment of mCRC remain major challenges in oncology. Therefore, early diagnosis of CRC metastasis and identification of new therapeutic targets before distant organ metastasis occur are crucial. Locoregional spread, a harbinger of distant metastasis, is a significant prognostic indicator, yet it exhibits biological heterogeneity. LNM is recognized as one of the significant prognostic factors in CRC. It was previously widely believed that distant organ metastasis was a direct consequence of LNM, however, increasing evidence suggests that approximately 65% of distant metastases are unrelated to LNM (21). Meta-analyses confirm CRC has other regional spread mechanisms with significant prognostic value, among which TD has the most profound prognostic impact (4). Clinically, TD has independent prognostic value for distant metastasis.

Research on TD has primarily focused on their independent prognostic value in clinical settings (8). Brouwer et al. (11), through spatial transcriptomics and proteomics analyses, revealed that at the level of regional metastasis, TD exhibits stronger stromal and fibrotic characteristics, as well as heightened immunosuppression, compared to LNM. Compared to prior studies, our research exhibits notable novelty in several aspects: Firstly, it is the first to investigate, using DIA-MS on FFPE specimens, alterations in protein expression profiles and pathways across different tissue types in N1c stage CRC, a subtype with significant prognostic controversy. Secondly, it identified a TD-associated metastatic risk gene signature and constructed a multi-cohort-validated TDRG model, providing a precise molecular tool for prognostic assessment in N1c CRC patients. Additionally, through integrated analysis of the immune microenvironment and immunotherapy biomarkers, we provide evidence to support the stratification of CRC patients for tailored immunotherapy. Preliminary single-cell localization, clinical correlation analysis, and functional validation of the TDRG PLOD1 have revealed its involvement in ECM remodeling and promotion of tumor migration and invasion in CRC. These findings not only enhance our understanding of the biological characteristics of TD but also provide novel tools and perspectives for clinical decision-making.

Previous studies have shown that TDRGs play a significant role in tumor etiology. We found that TDRGs are mainly enriched in the ECM remodeling and EMT pathway. The ECM is a complex network of biomolecules in the extracellular space that provides structural and mechanical support to surrounding cells. Normal ECM undergoes dysregulation of growth factors and enzyme levels transitioning towards a fibrotic and sclerotic cancerous phenotype (22–24). This transformation promotes tumor metastasis, the recruitment of CAFs and TAMs, and regulates immune responses (22, 25). Collagen (COL), an important component of the ECM, is mainly produced by CAFs. The crosslinking of COL in the ECM regulates the stiffness of tumor tissue, affecting tumor migration, invasion, and adhesion (26, 27). Among them, COL6A2 is one of the genes encoding the three alpha chains of type VI collagen. Studies have shown that COL6A2 can inhibit wound healing and invasion by inducing cell cycle arrest in the G1 phase and inhibiting the activity of matrix metalloproteinases MMP-2 and MMP-9 (28), thereby suppressing tumor metastasis. Additionally, research has found that an ECM rich in COL3A1 induces tumor dormancy, and the activation of DDR1/STAT1 triggers the expression of COL3A1 to maintain the tumor in a dormant state (29). Integrins are transmembrane receptors that mediate the interaction between ECM and cells. Their abnormal activation promotes tumor metastasis (30). Integrin alpha-2 (ITGA2) is overexpressed in CRC and has been identified as a marker for liver metastasis in CRC (31). It also plays a crucial role in enhancing 5-fluorouracil resistance in CRC (32). Fibromodulin (FMOD) is a leucine-rich small proteoglycan in the ECM (33) which promotes ECM remodeling by binding to lysyl oxidase (LOX). It has been reported that FMOD can induce tumor cell migration by enhancing the formation of stress fibers composed of filamentous actin (34).

ECM fibrosis is associated with metastasis and poor cancer prognosis. Specifically, fibrosis promotes cancer dissemination by enhancing integrin mechanosensory pathways, which activate EMT (35). The fibrotic process is primarily mediated by increased COL deposition and enhanced expression of ECM remodeling enzymes (36). Lysine hydroxylase 1, encoded by PLOD1, is a key ECM remodeling enzyme regulating collagen post-translational modification, cross-linking, and deposition (37). PLOD1 is implicated in the onset and metastasis of multiple tumors, including breast cancer where it promotes proliferation and distant metastasis by regulating collagen cross-linking (38). Overexpression of PLOD1 in bladder cancer correlates with poor prognosis (39), its mutations or overexpression have been found in gastrointestinal cancer (40). Furthermore, HIF-1 activates PLOD1 transcription under hypoxic TME, enhancing ECM stiffness through collagen hydroxylation (41). However, existing studies have not explored the function of PLOD1 in CRC. Using the TISCH 2.0 dataset, we analyzed PLOD1 expression and distribution at the single-cell level in CRC, revealing enrichment in CAFs, TECs, and malignant tumors. CCI analysis reveals their close interaction with malignant cells. Meanwhile, we validated the expression pattern of PLOD1 in N1c CRC tissues via IHC, and Masson’s trichrome staining further demonstrated a significant correlation between the increased collagen fiber abundance in TD and distant metastatic tissues and the expression level of PLOD1.

In TDRGs, genes related to metabolism (PGD, NDUFA8) and endoplasmic reticulum membrane, extracellular vesicles (HSPH1, SEC31A, RTCB) were also identified. 6-Phosphogluconate dehydrogenase (PGD) promotes glycolysis and fatty acid synthesis by inhibiting the AMPK signaling pathway, thereby driving tumor progression (42). NADH: ubiquinone oxidoreductase subunit A8 (NDUFA8), a subunit of mitochondrial complex I, is highly expressed in tumors, promoting mitochondrial respiration to support proliferation and inhibit apoptosis in cancer (43). The RNA ligase RTCB has a potential role in cancer, with its expression correlated with decreased DFS and OS in patients, and it mediates high resistance of breast cancer to anti-estrogen therapy (44). The coat protein complex II (COPII) transports COL from the endoplasmic reticulum to the Golgi apparatus (45), SEC31A, as an outer component of COPII, is involved in COL secretion. Its ubiquitination induces COL secretion, mediating the first step of ECM remodeling (46). HSPH1, a key member of the HSP70 superfamily, is significantly upregulated in CRC and associated with the sustained activation of Wnt and NF-κB signaling pathways (47–49). HSPH1 can be actively secreted by cancer cells into the ECM, promoting the polarization of macrophages towards the M2 phenotype (50). TDRGs may be closely related to the pathogenesis and progression of CRC, but our study is the first to explain their correlation with the risk of distant metastasis in N1c CRC and to develop a combined prognostic indicator.

Our drug sensitivity analysis revealed that high-risk CRC cases exhibit poor responses to conventional chemotherapy agents but demonstrate relative sensitivity to BI2536, dasatinib, and staurosporine. Notably, the biomechanical properties of the ECM represent a critical mechanism underlying chemotherapy resistance. Studies have indicated that metastasis-associated fibroblasts (MAFs) can diminish drug efficacy in CRC liver metastases by modulating ECM stiffness (51). For the PLK1 inhibitor BI2536, it exhibits antifibrotic advantages, significantly impacting primary fibroblasts (52). Furthermore, the PLK1 inhibitor onvansertib, in combination with FOLFIRI and bevacizumab, has demonstrated favorable efficacy and tolerability in second-line treatment of KRAS-mutant mCRC (53). Additionally, the Src/ABL inhibitor Dasatinib has shown efficacy in paclitaxel-resistant, Trp53/Cdh1-deficient mouse gastric adenocarcinoma with peritoneal metastasis by inhibiting ECM-receptor interaction signaling pathways (54). Given that our current findings are based on predictions from cell line models, subsequent research could focus on establishing a biobank of patient-derived organoids (PDOs) from N1c CRC or high TDRG scores patients to further validate the sensitivity of these drugs and explore their synergistic antitumor effects in combination therapies.

In the realm of CRC immunotherapy, microsatellite-stable (MSS) CRC, which accounts for the majority of CRC cases, often exhibits a low response to immune checkpoint inhibitors (ICIs) due to complex immunosuppressive mechanisms. Currently, combination therapies represent the primary avenue of exploration (55–57). Notably, CMS4 and N1c subtypes of CRC, which may exhibit a higher degree of stromal infiltration, demonstrate more pronounced and direct immune exclusion, with the ECM barrier playing a pivotal role (58). Transforming growth factor-beta (TGF-β) fosters a fibrotic barrier by activating CAFs to secrete excessive ECM. TGF-β inhibitors can counteract immunosuppression and reduce the ECM barrier, thereby amplifying the efficacy of ICIs through an immune-enhancing effect. Dual-targeted agent that simultaneously inhibits two distinct immune escape pathways—PD-(L)1 and TGF-β—in the TME (59), has demonstrated clinical activity in patients with various malignancies, including metastatic cervical cancer (60), gastric or gastroesophageal junction adenocarcinoma (G/GEJA), and mCRC (61). Recent studies have also unveiled the dynamic regulation of the tumor-stroma boundary in pMMR/MSS CRC. Feng et al. (62) discovered that boundary tumor cells in pMMR CRC drive the differentiation of CXCL14^+^CAFs via the IHH/PTCH1 axis, forming a physical ECM barrier. Wu et al. (63) revealed that CAFs at the invasive front of MSS CRC secrete chondroitin-6-sulfate (C-6-S) to induce macrophage polarization, thereby shaping an immune exclusion barrier. These findings offer potential strategies for targeting the ECM barrier—reprogramming the tumor-stroma boundary to enhance ICI efficacy. Future combination strategies may leverage these unique mechanisms to reshape the TME, break immunosuppression, and provide hope for overcoming the immunotherapy bottleneck in N1c CRC.

We acknowledge several limitations in this study that point to important directions for future research. First, the inclusion of only 13 patients, combined with the small sample size and absence of stratified analysis, may have compromised the robustness of our findings. The lack of spatial multi-region sampling also obscured intratumoral spatial heterogeneity. Furthermore, the TDRG model was trained and validated in mixed-stage CRC transcriptomic datasets, although its constituent genes were derived from an N1c-specific proteomic landscape. To address these limitations, we plan to expand sample size via multi-center collaboration for non-pooled omics analysis of N1c CRC and retrospectively validate the TDRG model in more homogeneous Stage III subpopulations and CRC-specific ICI treatment cohorts. Secondly, due to practical constraints, we employed composite samples from analogous tissues of different patients for DIA-MS. Although we adopted a stratified randomization and batch-balanced sample pooling strategy, the lack of individual patient-level proteomic data undermines the precise correlation between proteomic signatures and clinicopathological features. Given the heterogeneity among CRC patients, pooled samples may overlook key proteins or pathways associated with specific subtypes. Subsequent studies will focus on non-pooled individual sample analysis to enhance the resolution of proteomic profiling. Thirdly, while the use of multiple algorithms improves model flexibility, it also increases the risk of overfitting and algorithm selection bias. In future work, we will simplify the algorithms used for feature extraction and model construction to reduce overfitting and will explore ensemble learning approaches to improve generalization by combining weak learners. Moreover, although we have established a functional and correlative role for PLOD1, the exact molecular mechanisms through which it drives metastasis remain to be fully elucidated. We will further employ *in vitro* collagen cross-linking assays, detailed analysis of EMT and integrin pathways following PLOD1 modulation, and *in vivo* experimental/spontaneous metastasis models to comprehensively uncover the role of PLOD1 in driving N1c CRC metastasis. Lastly, to further explore the biological traits and specificities of N1c entities, we are integrating single-cell transcriptome sequencing with spatial transcriptomics to analyze N1c (TD+) samples and compare them with lymph node metastasis CRC samples. This approach aims to uncover distinct microenvironment remodeling patterns and cellular interaction mechanisms across different regional metastatic pathways.

## Conclusion

We assessed the protein expression profiles and pathway alterations of N1c CRC samples. The model developed based on machine learning-identified TDRGs, effectively predicted DFS and immunotherapy response in external dataset, supporting precision treatment and staging system optimization. PLOD1’s role in ECM remodeling and CRC cell migration and invasion suggests its potential as a prognostic biomarker and therapeutic target.

## Data Availability

The datasets presented in this study can be found in online repositories. The names of the repository/repositories and accession number(s) can be found in the article/**Supplementary Material**.

## Supplementary material

Supplementary Table 1 Demographic and clinicopathological characteristics of CRC patients for proteomics analysis.

Supplementary Table 2 Inter-observer variability data from consistency analysis in pathological assessment.

Supplementary Table 3 Four additional modules (1, 2, 4, 5) identified within the PPI network using MCODE via Cytoscape, along with their enrichment analysis results.
