# Supplementary material for: Mass spectrometry-based multi-omics analysis elucidates immune microenvironmental characteristics and the risk of distant metastasis in N1c colorectal cancer
Source: Front Immunol. 2026 Feb 17;17:1590042. doi: 10.3389/fimmu.2026.1590042 (PMC12953441; doi:10.3389/fimmu.2026.1590042)
Supplement: Supplementary Table 3 — Four additional modules (1, 2, 4, 5) identified within the PPI network using MCODE via Cytoscape, along with their enrichment analysis results. [file Table3.docx]

FFPE-based Proteomics and Machine Learning Reveal Immune Microenvironment Features and Distant Metastasis Risk in N1c Colorectal Cancer

Supplementary Material

Supplementary Figure S1：

The evaluation of Quality Control (QC) samples and the DIA system, covering metrics such as the Coefficient of Variation (CV), indexed Retention Time（iRT）, full width at half maximum (FWHM), and peptide length distribution.

**S1A.** Distribution plot of CV values for QC (X-axis: Range of CV values for QC; Y-axis: Number of QC samples within each CV interval.)


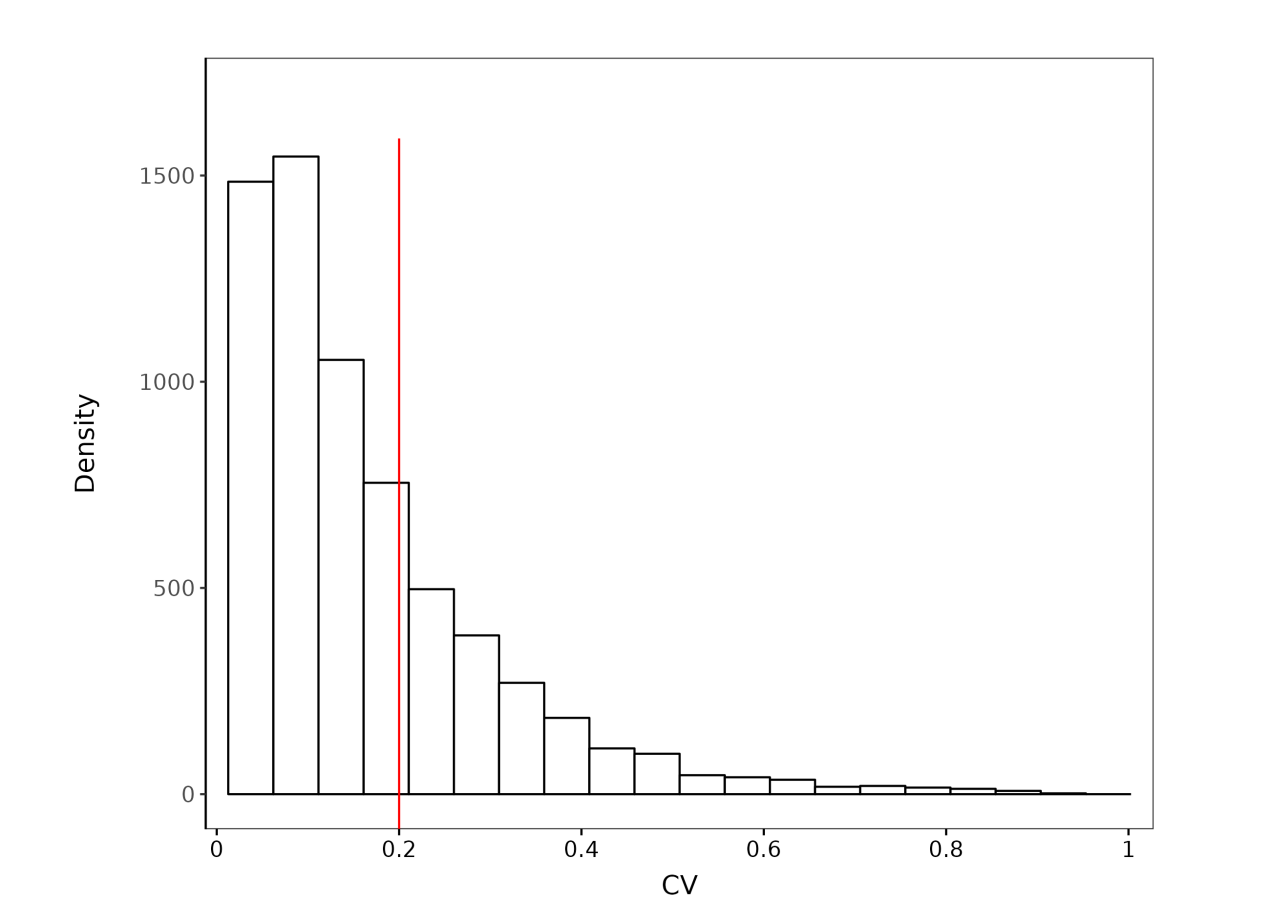


**S1B.** The elution time data of each peptide segment from the iRT Kit on the chromatography in this project，the main iRT peptides were all detected, and their retention times were generally stable.


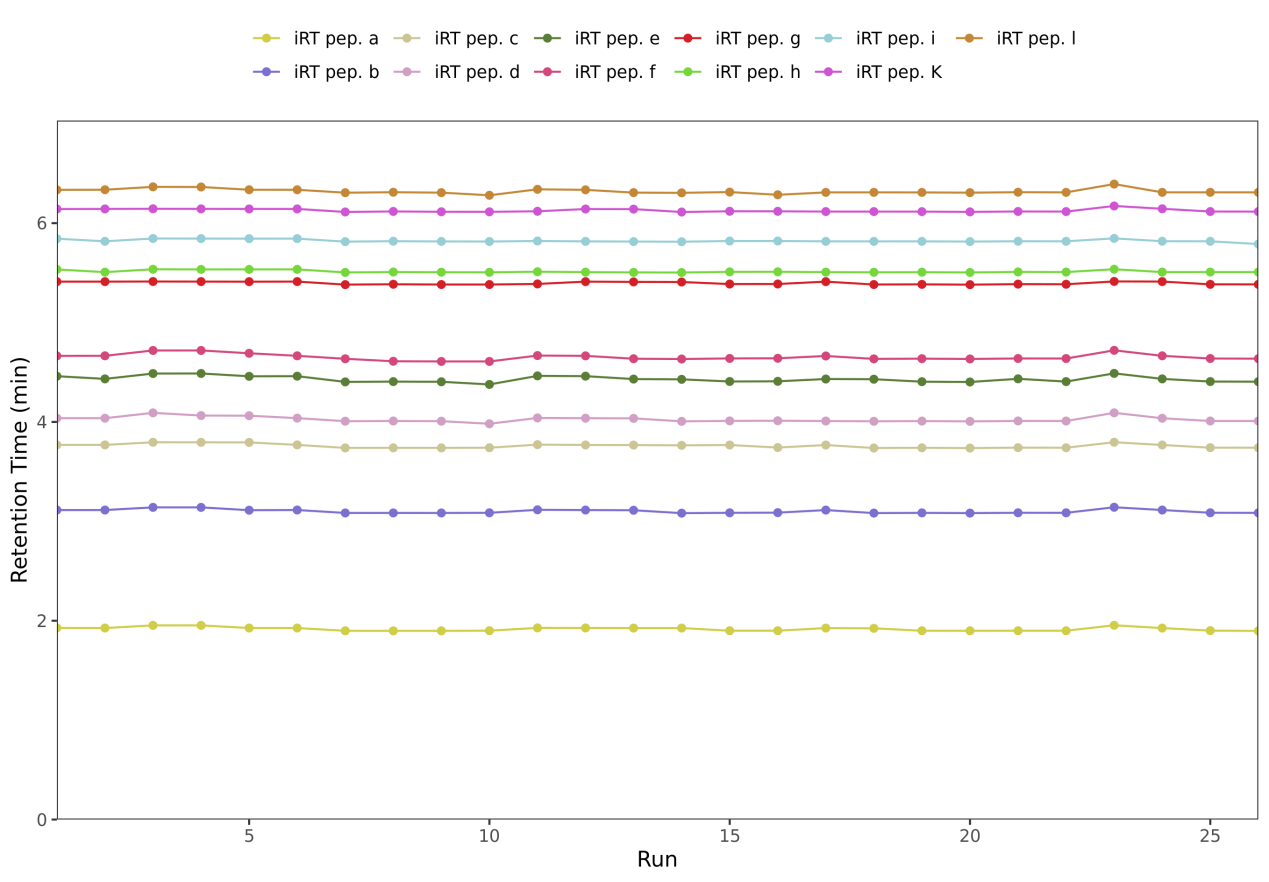


**S1C.** The fluctuation of the average FWHM in this project.


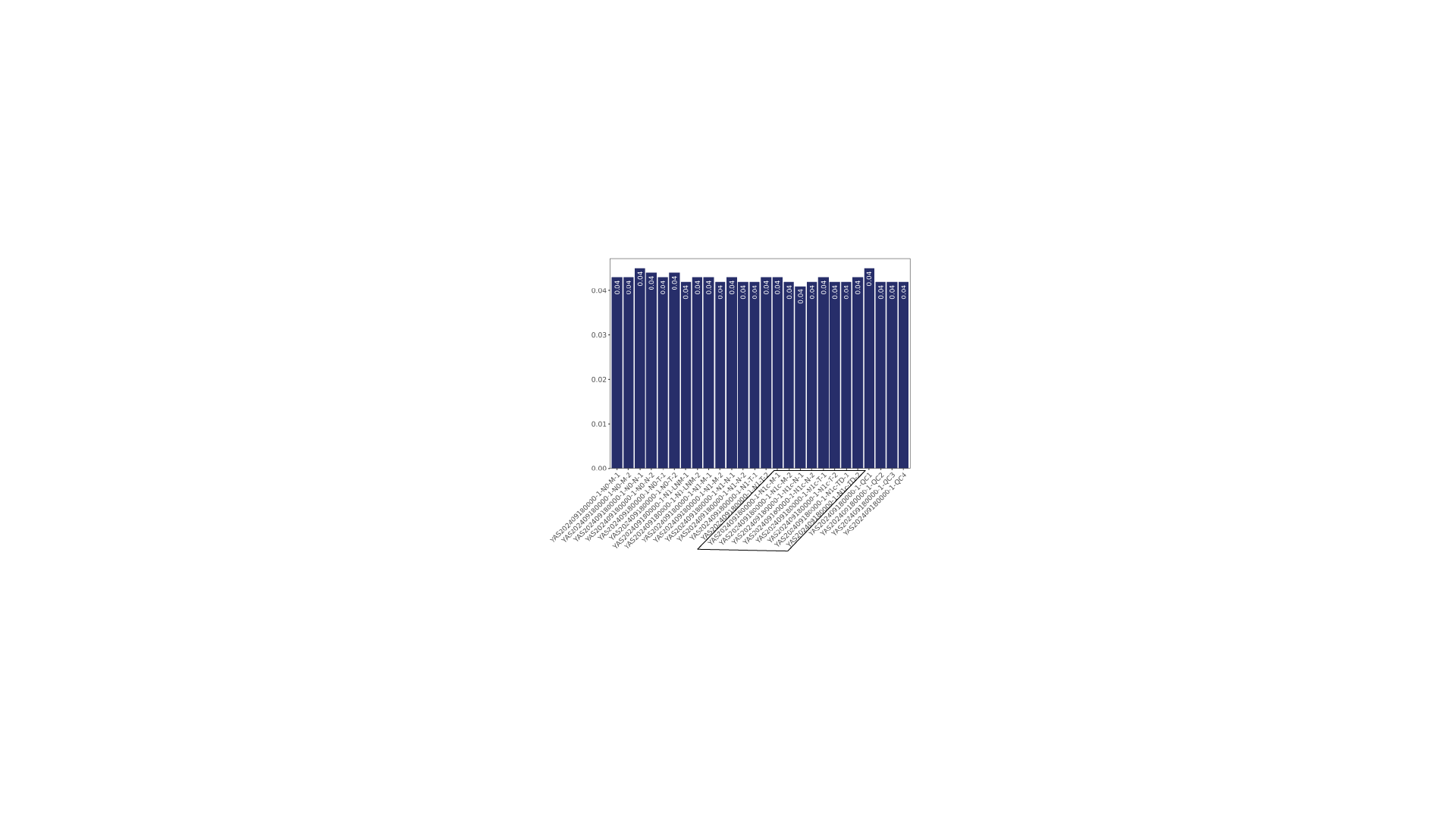


**S1D.** Distribution plot of peptide sequence lengths


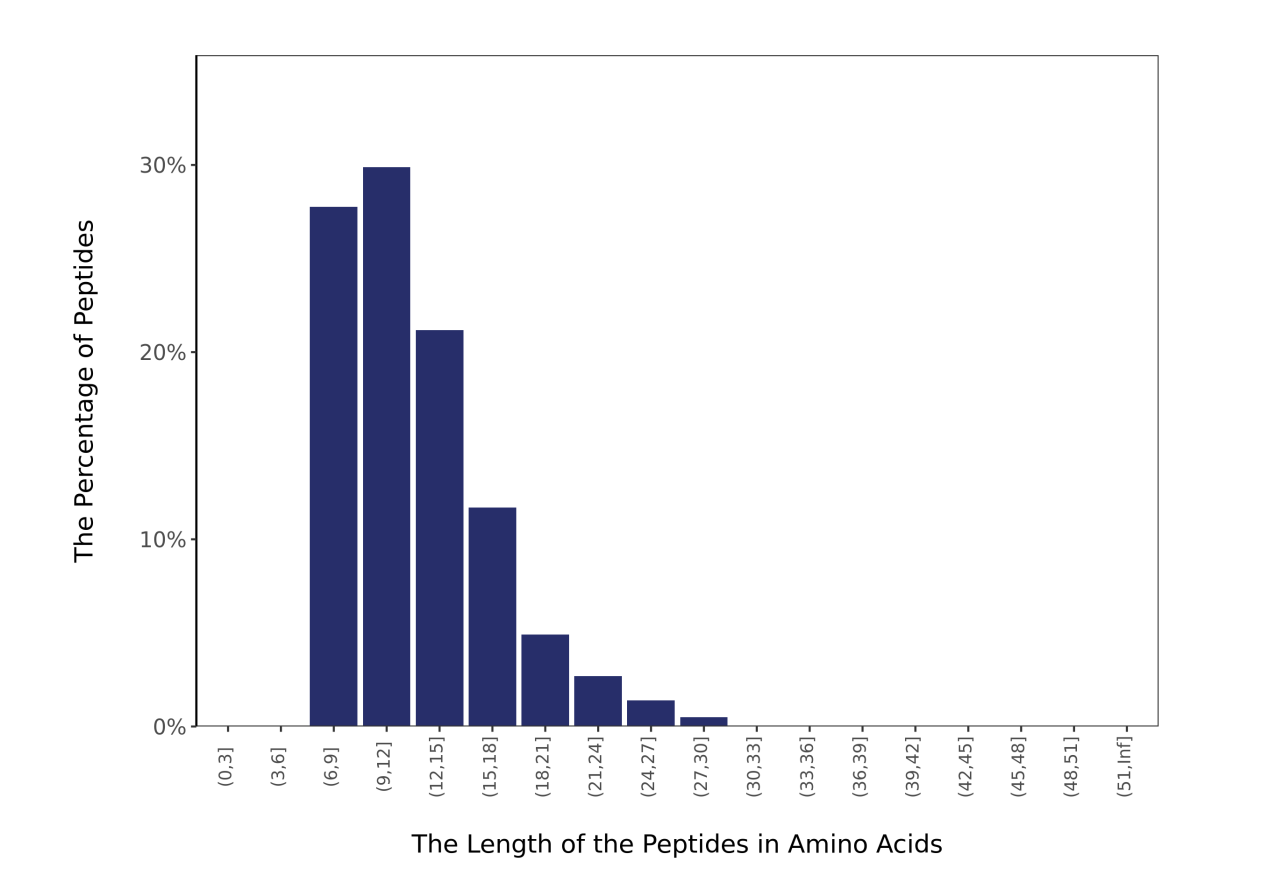


Supplementary Figure S2：

GO and KEGG enrichment analysis of differentially expressed proteins among normal tissue vs. primary tumor, TD, and liver metastases.

Methods：Kyoto Encyclopedia of Genes and Genomes (KEGG), Gene Ontology (GO), and Gene Set Enrichment Analysis (GSEA) using the "ClusterProfiler" package and the GSEA software (<https://www.gsea-msigdb.org/gsea/index.jsp>). The h.all.v7.4.symbols.gmt subset was downloaded from the Molecular Signatures Database (MSigDB, <https://www.gsea-msigdb.org>) to assess the correlation between gene expression levels and biological pathways or molecular mechanisms.

**A.** GO_CC/BP/MF enrichment of differentially expressed proteins (Normal vs. Tumor)


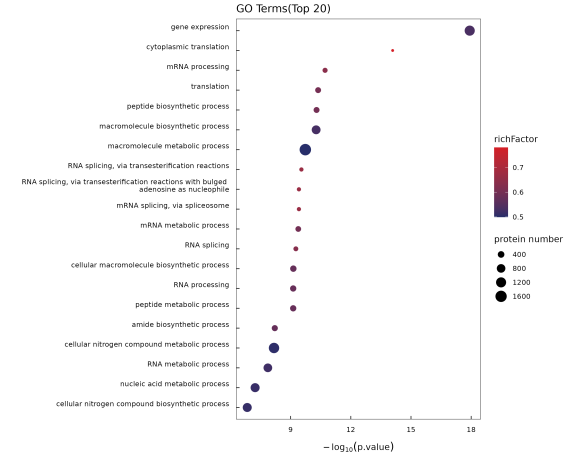

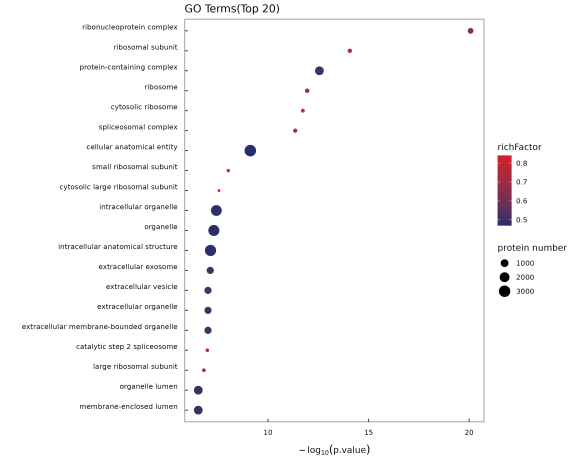


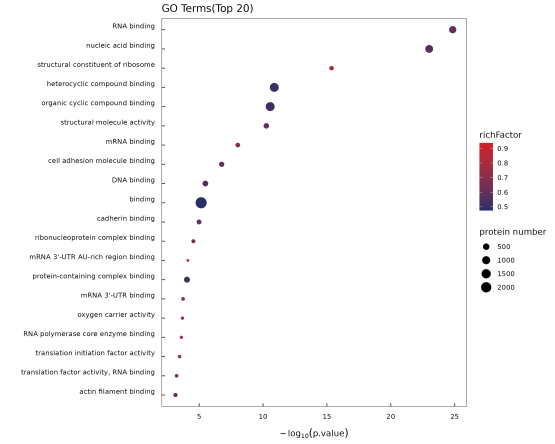


**B.** KEGG enrichment of differentially expressed proteins (Normal vs. Tumor)


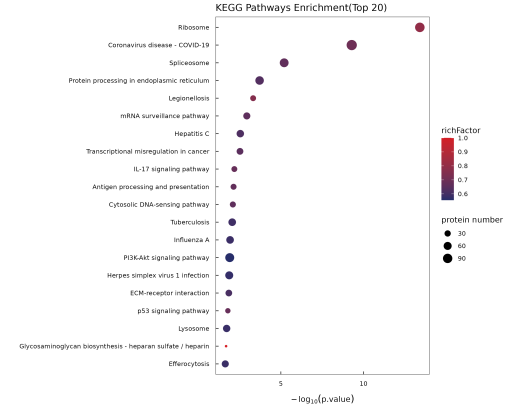


**C.** GO_CC/BP/MF enrichment of differentially expressed proteins (Normal vs. Tumor deposit)


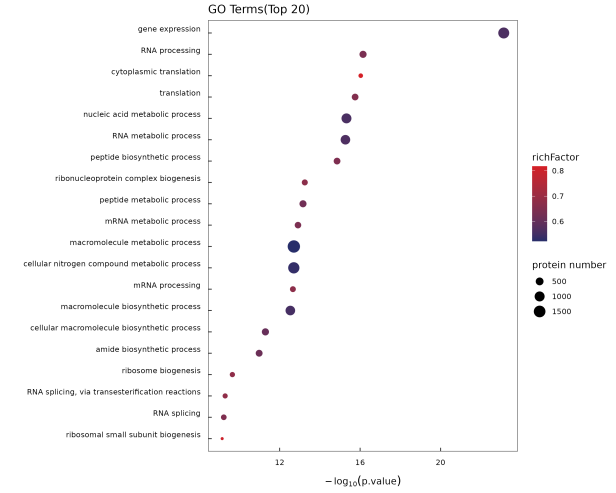

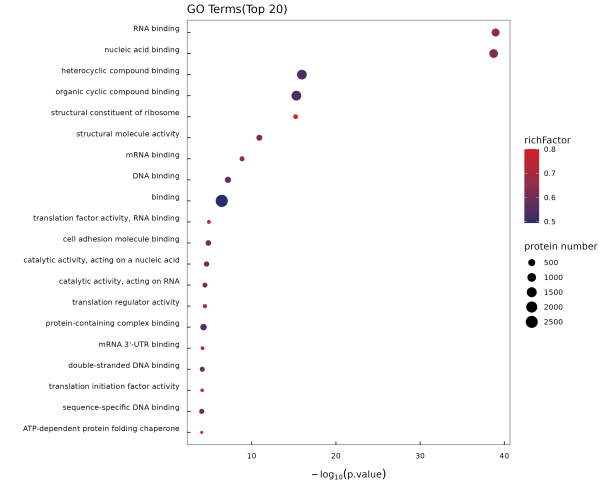

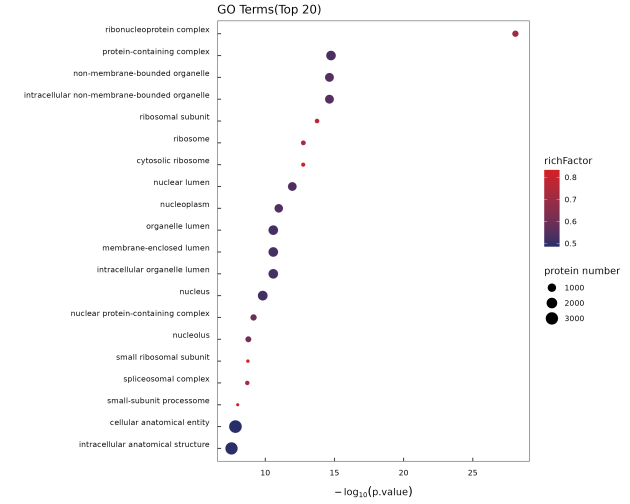


**D.** KEGG enrichment of differentially expressed proteins (Normal vs. Tumor)


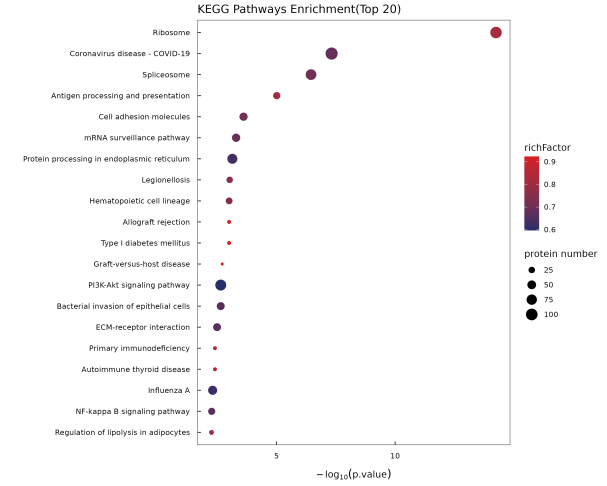


**E.** GO_CC/BP/MF enrichment of differentially expressed proteins (Normal vs. Metastasis)

**
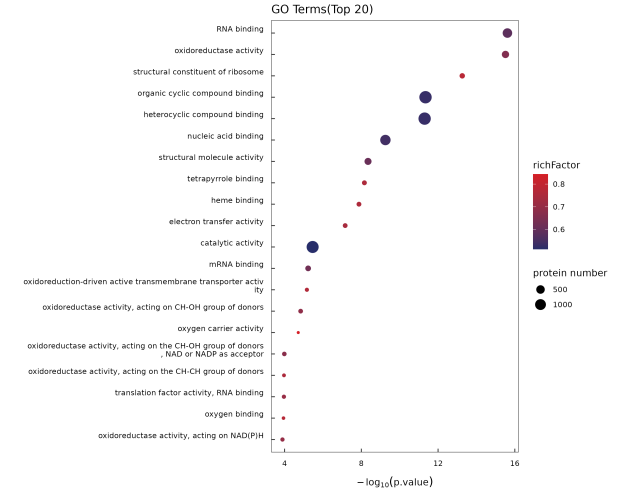

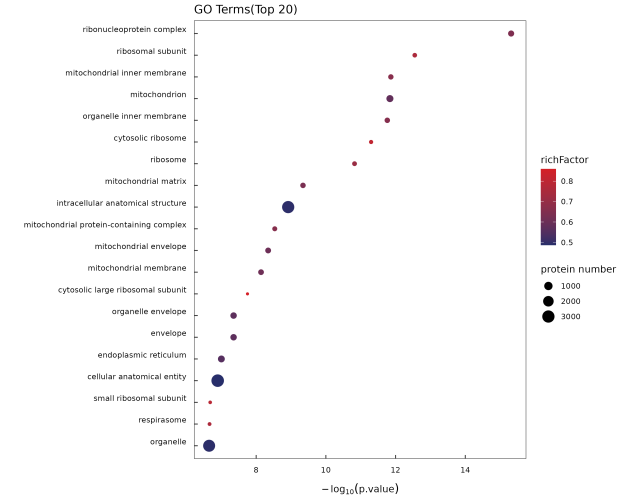
**


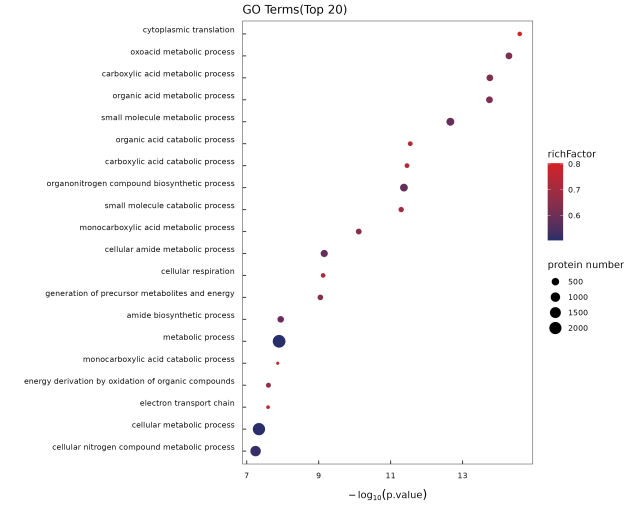


**F.** KEGG enrichment of differentially expressed proteins (Normal vs. Tumor)

**
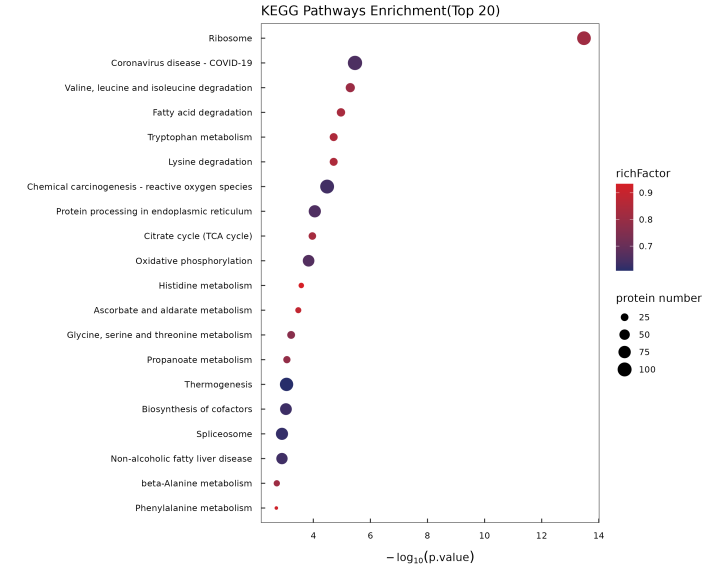
**

Supplementary Figure S3：

The Decision Curve Analysis (DCA) curves of the TDRG model in the training cohort (CCRC) and validation cohort. (GSE33113, GSE38832, GSE39582)

Methods：For both the training set and external cohorts, the rmda package was utilized to conduct DCA for the TDRG model to demonstrate net benefit.

**A**：The DCA curve of the TDRG model in the training cohort CCRC.


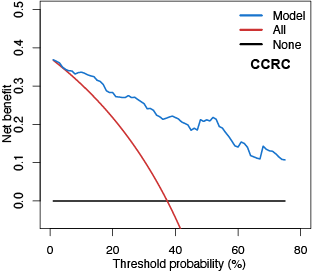


**B**：The DCA curve of the TDRG model in the validation cohort GSE33113.


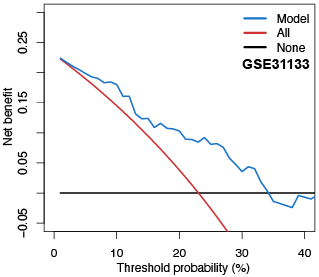


**C：**The DCA curve of the TDRG model in the validation cohort GSE38832.


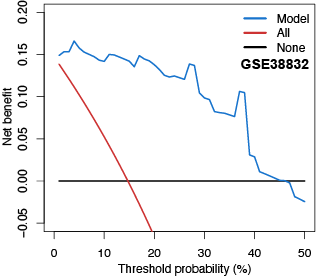


**D：**The DCA curve of the TDRG model in the validation cohort GSE39582.


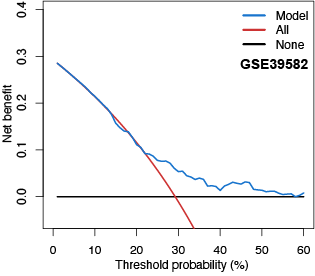


Supplementary Figure S4：

The associations between the RiskScore and MMR/KRAS/BRAF/TP53 status.

Methods: The Wilcoxon rank-sum test was used to compare the differences between groups to reveal the correlation between TDRG risk score and diverse molecular characteristics.

**A:** The associations between the RiskScore and tumor location.


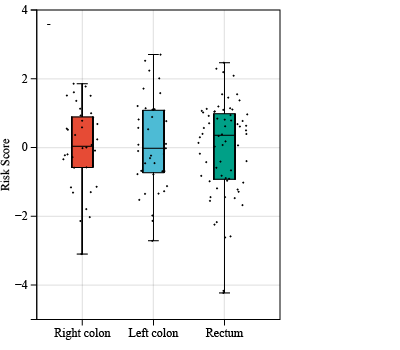


**B:** The associations between the RiskScore and MMR status.


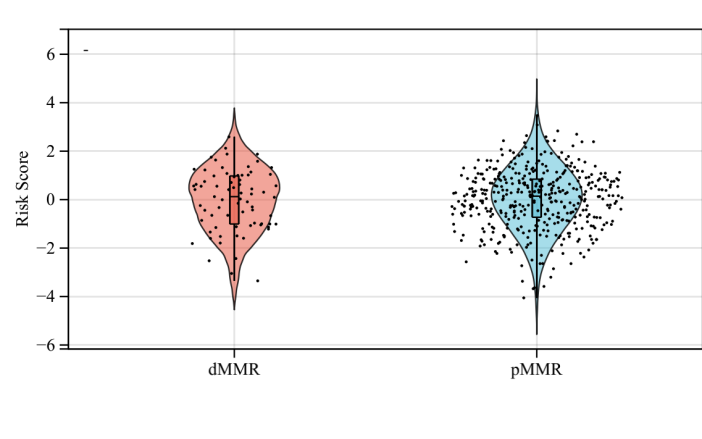


**C:** The associations between the RiskScore and KRAS status.


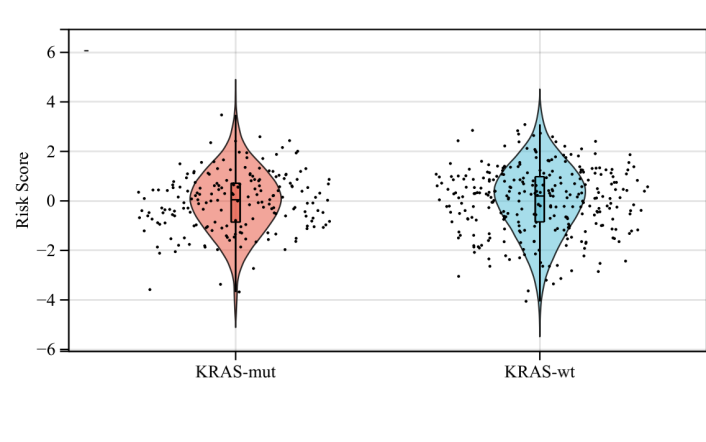


**D:** The associations between the RiskScore and BRAF status.

**
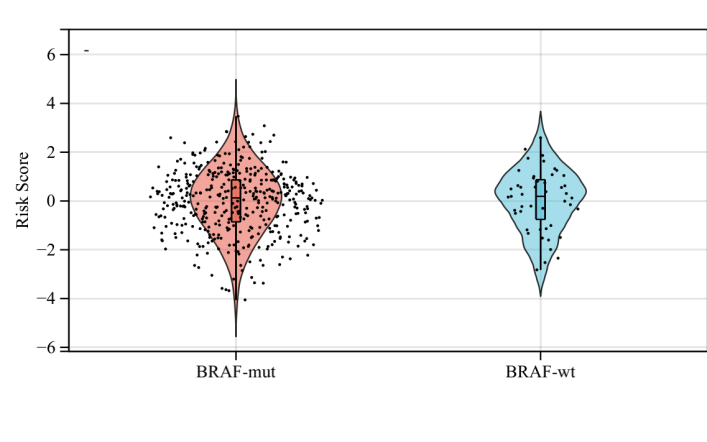
**

**E:** The associations between the RiskScore and TP53 status.


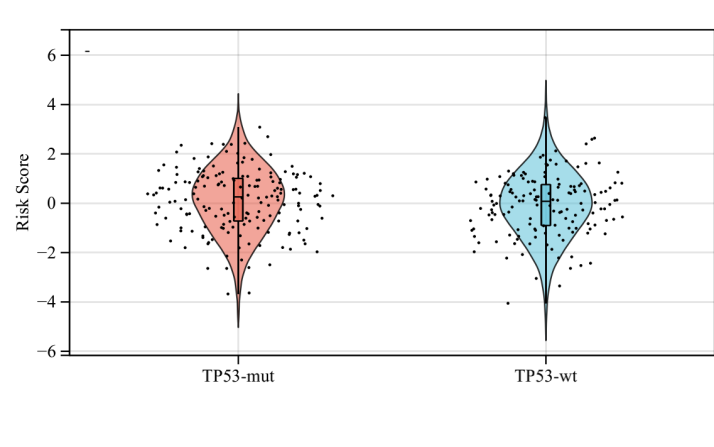


Supplementary Figure S5：

Mutation landscape across risk groups.

## Methods：We extracted mutation data from patients in the TCGA-COAD and TCGA-READ cohorts. Based on the TDRGs model, risk subgroups are classified. Utilize the "mafTools" R package to analyze somatic mutations in the high-risk and low-risk groups, as well as mutations in 10 TDRGs across all samples.

**A.** Mutation incidence of low-risk group.

**
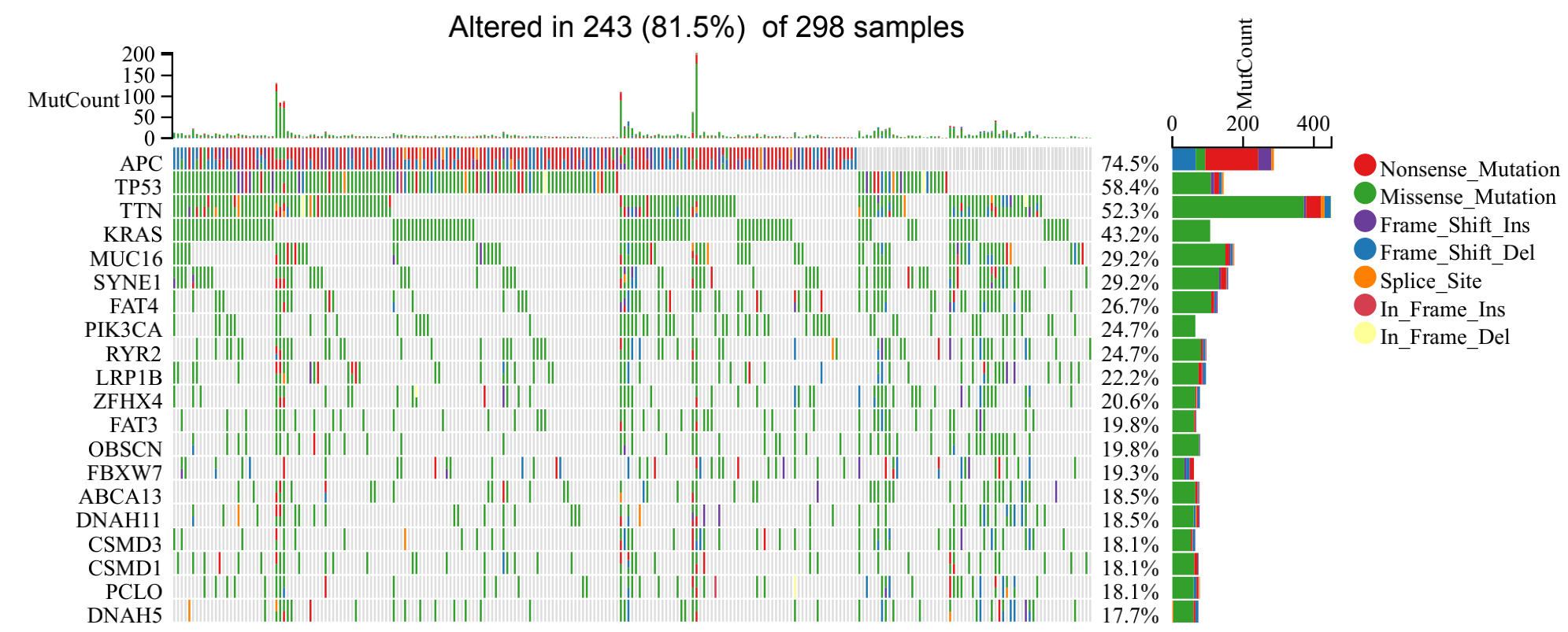
**

**B.** Mutation incidence of high-risk group.

**
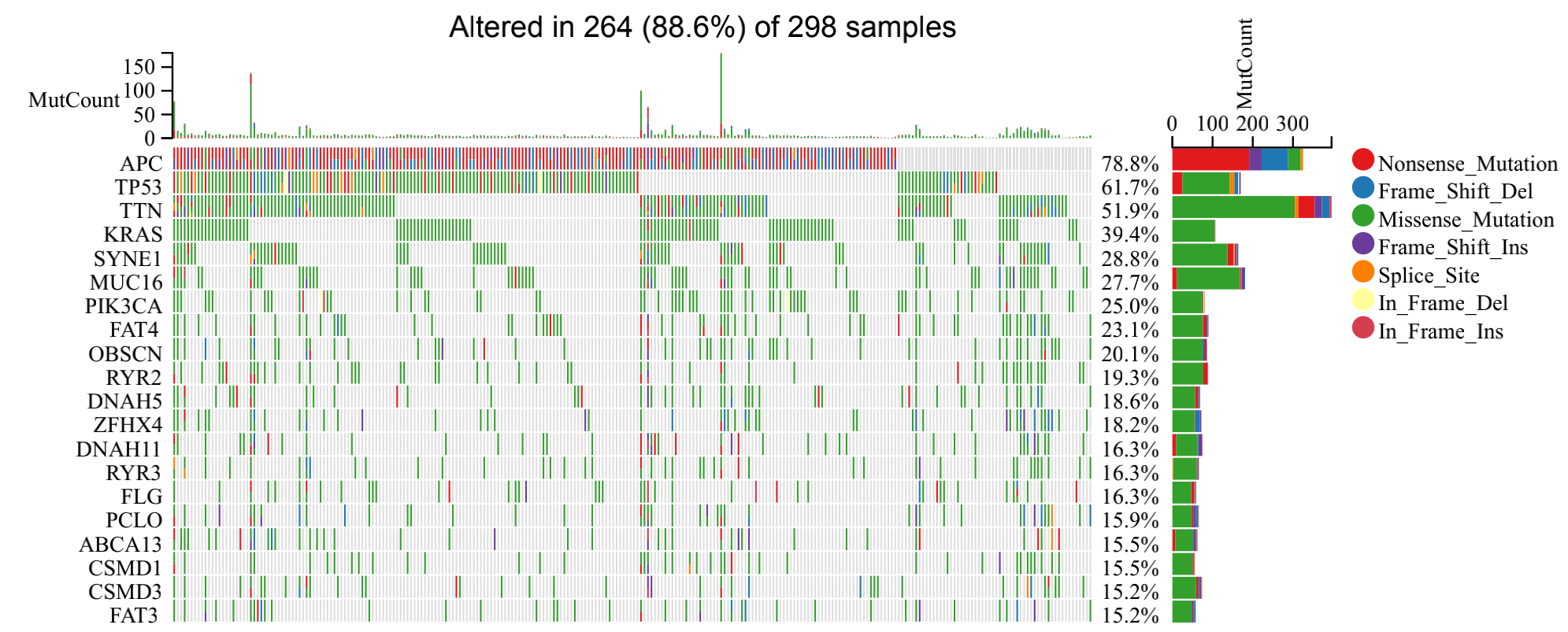
**

**C.** Mutation status of TDRGs in samples.


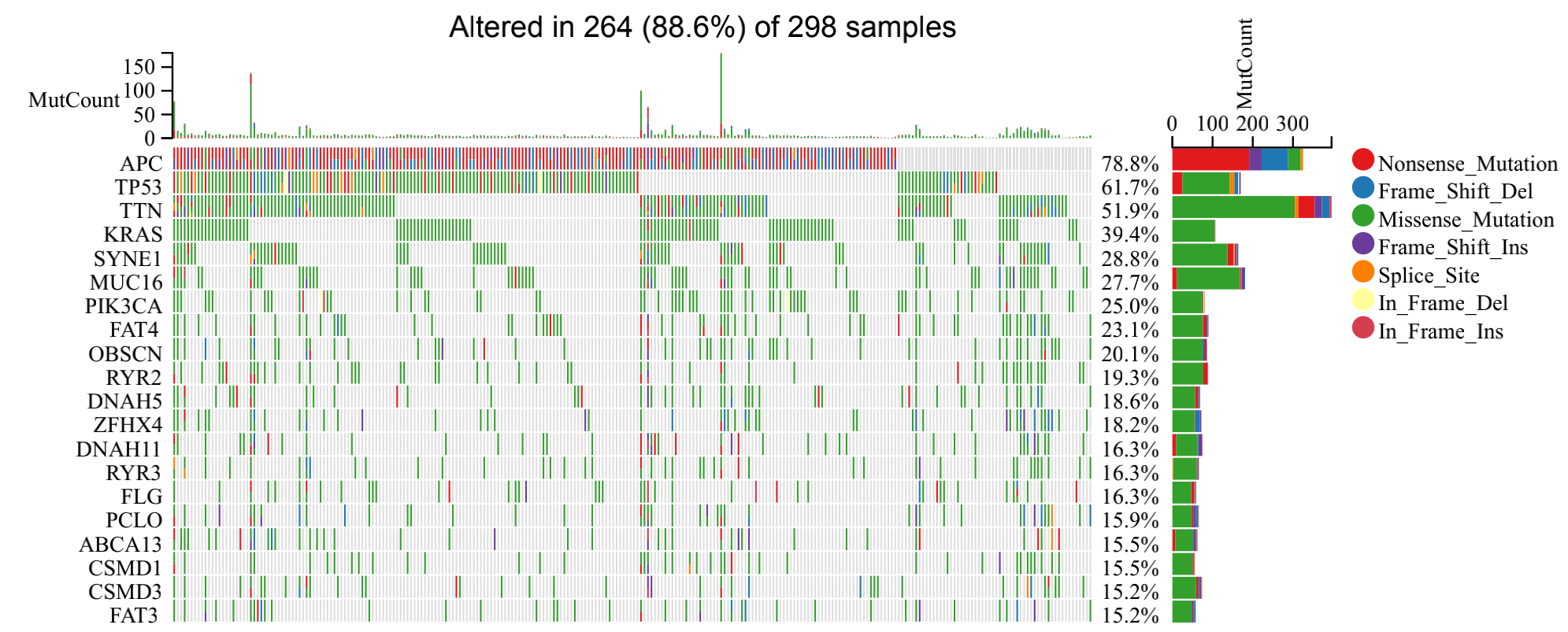


Supplementary Figure S6：

The results of GO and KEGG enrichment analyses of TDRGs.

**A:** GO enrichment of TDRGs


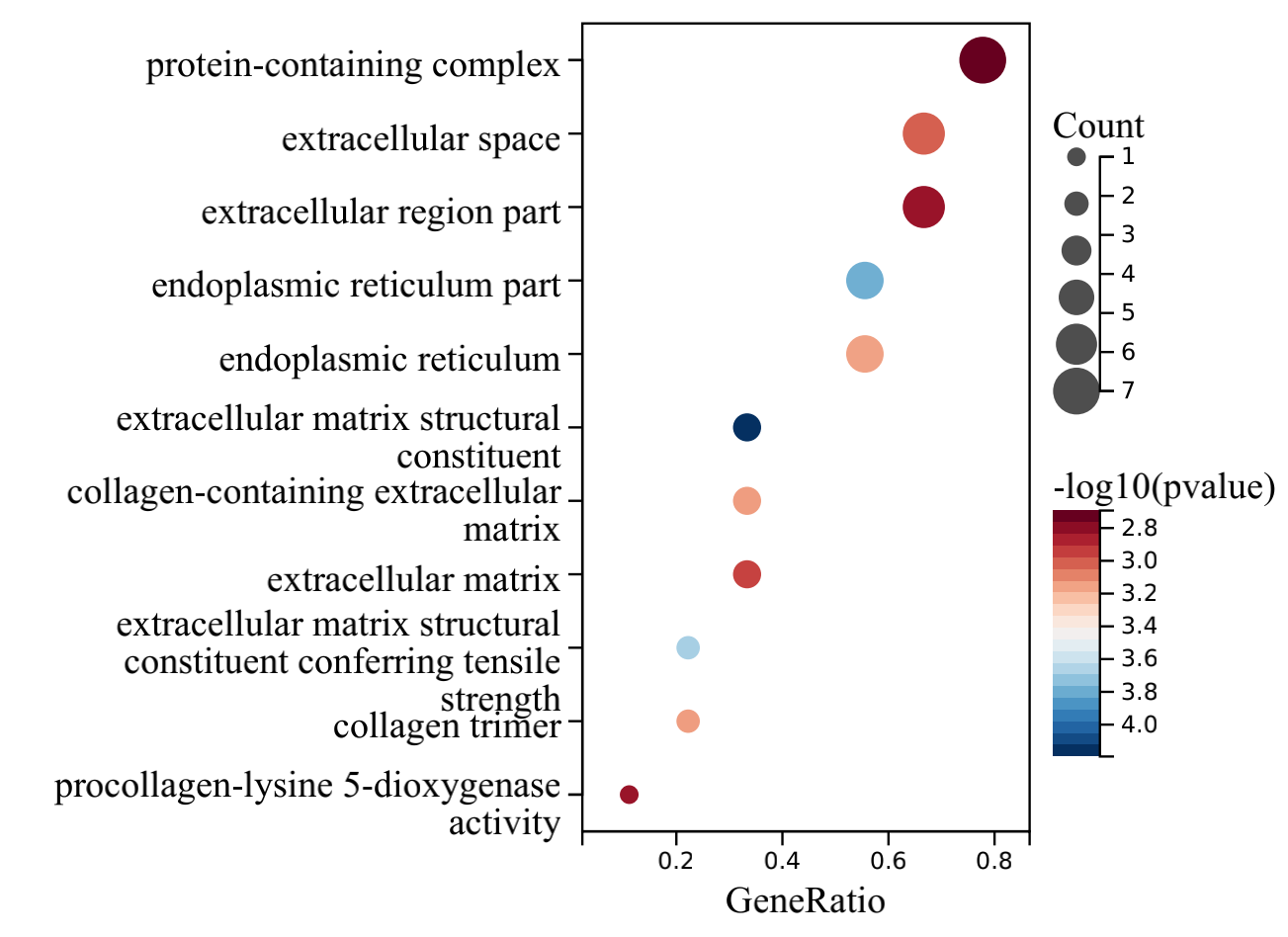


**B:** KEGG enrichment of TDRGs


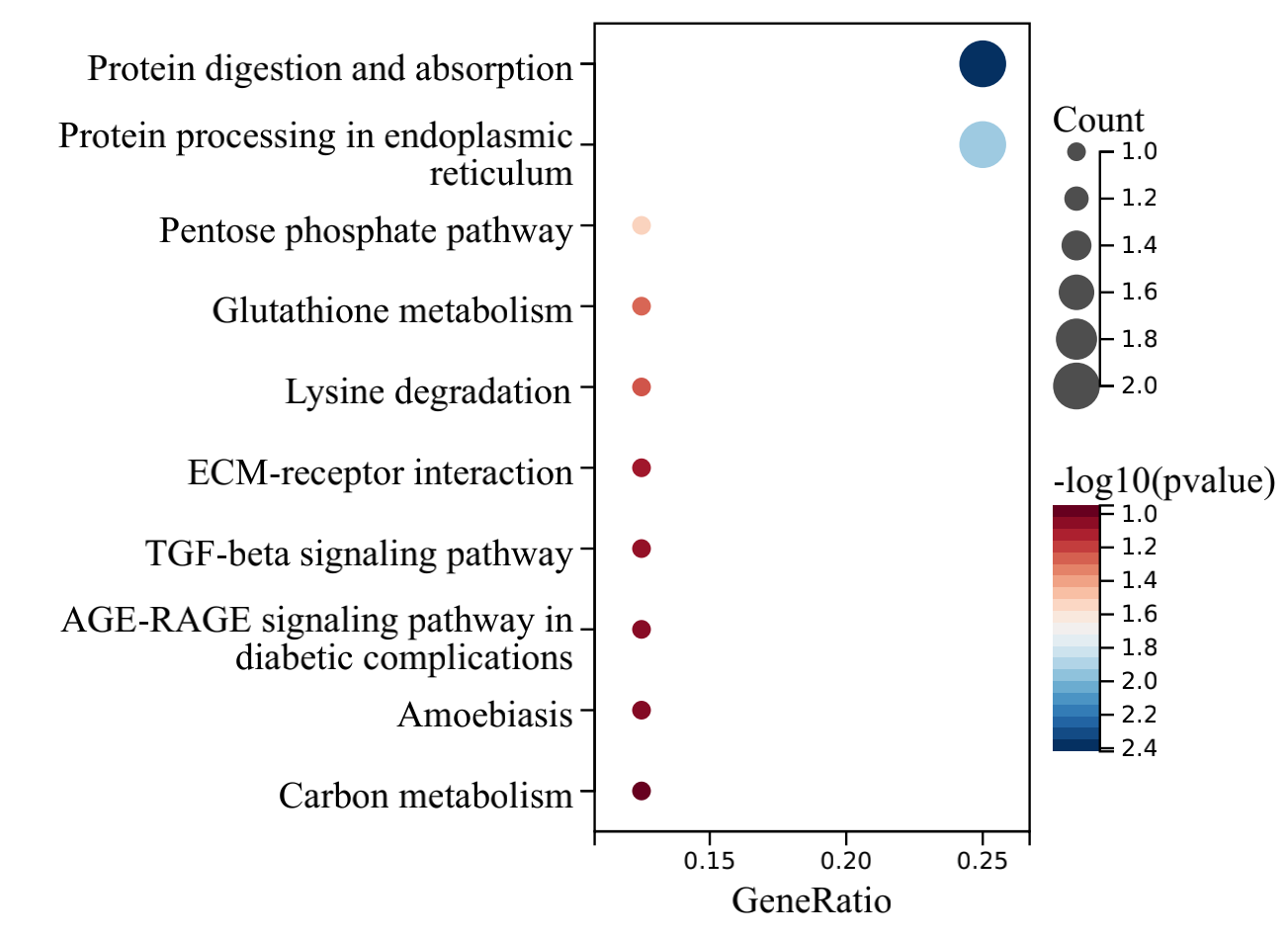


Supplementary Figure S7：

Non-parametric test results for key EMT-related gene expression across different risk groups

Methods: For more classic EMT-related genes, We conducted differential expression analysis across risk groups in multiple cohorts (GSE38832, GSE31133), the results revealed significant differences in CDH, FOXC2, TWIST1, TWIST2, SNAI2, ZEB1, and ZEB2.

**A:** Expression differences of key EMT genes among different risk groups in GSE38832.

**
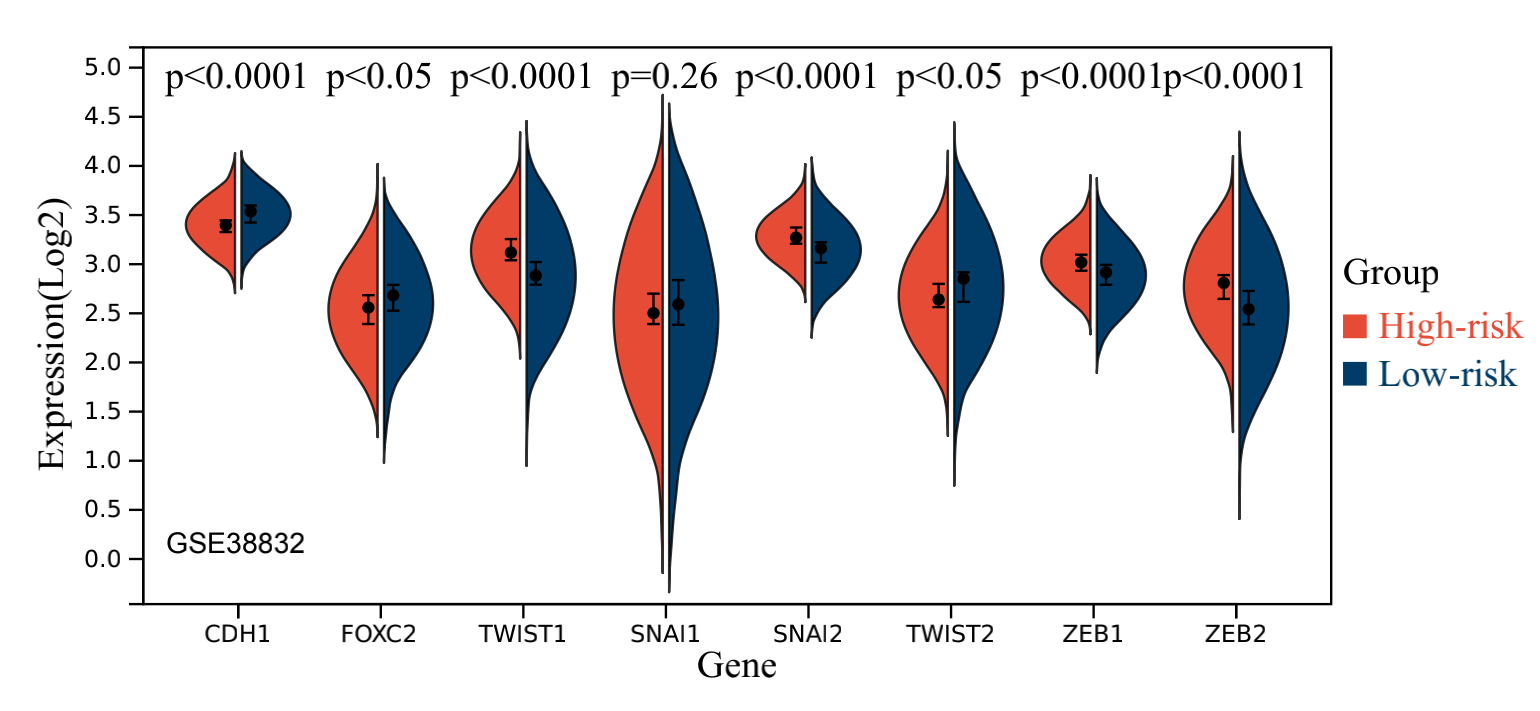
**

**B:** Expression differences of key EMT genes among different risk groups in GSE33113.**
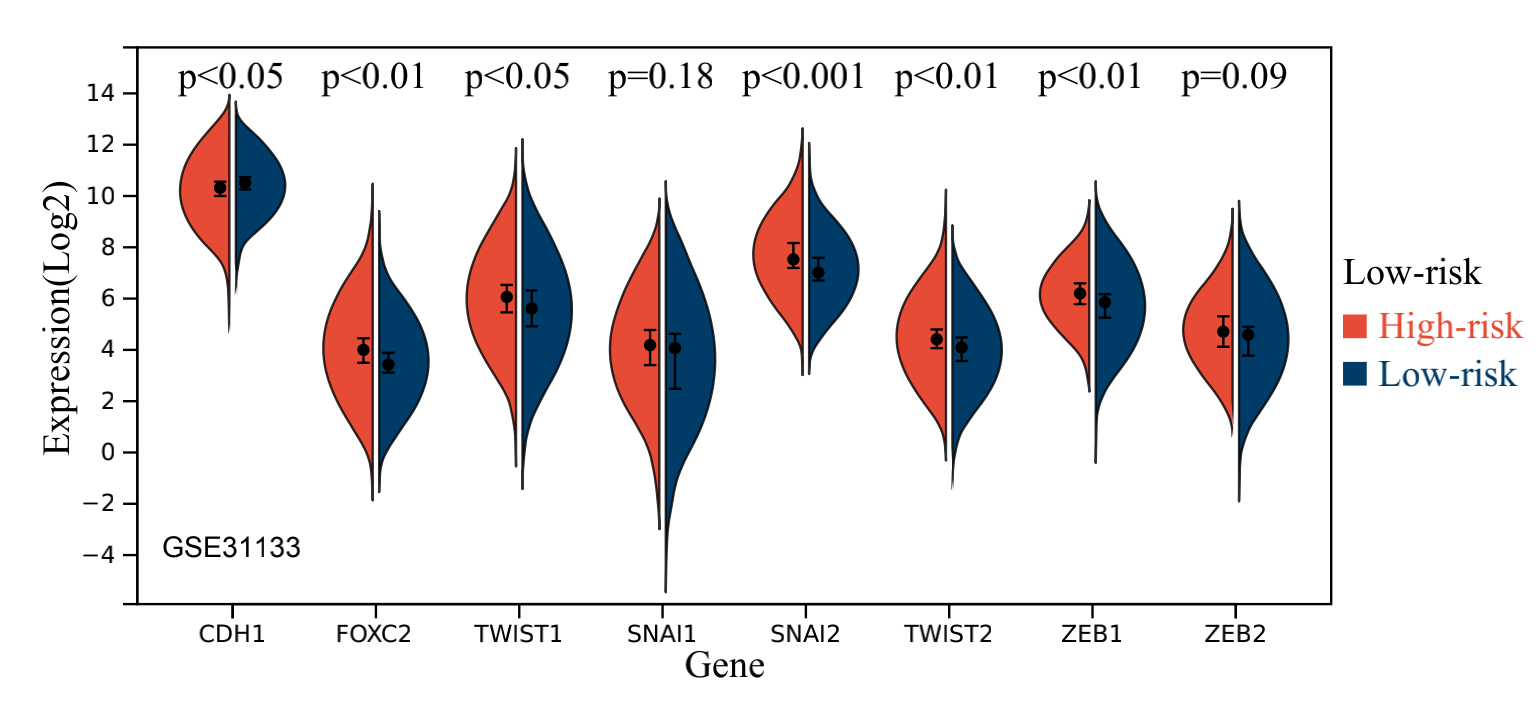
**
